# A Meta‐analysis of Functional Outcomes and Recovery Metrics Comparing Transoral Robotic Surgery and (Chemo)Radiotherapy

**DOI:** 10.1002/ohn.70069

**Published:** 2025-12-07

**Authors:** Simpson Shiu Chung Tam, Nihal Sogandji, Muzammil Arif Din Abdul Jabbar, Shazia Huma Absar, Mikesh Kalpesh Patel, Jeffrey Tooze, Eleanor Barker, Manaf Khatib, George Mochloulis, Anant Patel, Amit Gupta

**Affiliations:** ^1^ School of Clinical Medicine University of Cambridge Cambridge UK; ^2^ Medical Library University of Cambridge Cambridge UK; ^3^ Department of Plastic Surgery, Lister Hospital East and North Hertfordshire NHS Trust Stevenage UK; ^4^ Department of Otorhinolaryngology, Lister Hospital East and North Hertfordshire NHS Trust Stevenage UK; ^5^ Mount Vernon Cancer Centre East and North Hertfordshire NHS Trust Northwood UK

**Keywords:** head and neck cancer, head and neck surgery, oncology, oropharyngeal squamous cell carcinoma, robotic surgery, swallowing outcomes otorhinolaryngology, transoral robotic surgery

## Abstract

**Objective:**

To assess patient outcomes with regard to swallowing, feeding tube dependence, tracheostomy use, pain levels, and recovery metrics between transoral robotic surgery and (chemo)radiotherapy.

**Data Sources:**

Medline, EMBASE, Web of Science Core Collection, and the Cochrane Library.

**Review Methods:**

A meta‐analysis was conducted in accordance with the Preferred Reporting Items for Systematic Reviews and Meta‐analyses guidelines through a literature search of data sources (CRD42023464144).

**Results:**

Sixteen studies comprising 2185 patients were included. There were no significant differences in sex and age distribution between the transoral robotic surgery and (chemo)radiotherapy groups. Most patient demographics and disease status at baseline were comparable between the two groups. Meta‐analysis of the studies found reduced gastrostomy tube usage in the transoral robotic surgery group at 6 and 12 months albeit poorer swallowing outcomes at 12 months according to the MD Anderson Dysphagia Index. Pain levels were generally lower in the transoral robotic surgery group in the long term.

**Conclusion:**

This review presents the most extensive examination to date of swallowing performance and recovery metrics in controlled trials of transoral robotic surgery versus (chemo)radiotherapy. Our findings show that transoral robotic surgery shows promise in managing difficult anatomical areas and low‐grade tumors. However, methodological inconsistencies were noted between included studies, particularly in eligibility criteria and human papillomavirus p16 status. Consensus statements and/or guidelines on stratification and reporting of patients may aid in reducing this heterogeneity, while further randomized controlled trials may improve our understanding and guide the development of clinical guidelines on treatment choice.

The incidence of oropharyngeal squamous cell carcinoma (OPSCC) in North America and European countries has increased dramatically over the past decade.^1^, ^2^ OPSCC associated with infection with the high‐risk human papillomavirus (HPV)^2^ particularly HPV strain 16, accounts for 51.8% of all OPSCC cases in the United Kingdom.^3^ HPV‐associated OPSCC typically has a better prognosis than HPV‐negative OPSCC, the latter being more associated with risk factors such as smoking and alcohol consumption.^2^

The survival rates of OPSCC have also improved across decades, and whilst management may involve a combination of surgery, radiotherapy (RT), and chemotherapy, new treatments have now become available, including the use of transoral robotic surgery (TORS) for tumor removal. TORS offers increased precision and range of motion, with seven degrees of freedom compared to endoscopic instruments that have just four.^4^ Patients are thought to benefit from reduced pain, quicker recovery, shorter hospital stay, improved speech and swallowing, and minimal visible scars from small incisions.^5^ However, reported complications include postoperative hemorrhage, infection, dental trauma, and notably dysphagia.^6^, ^7^ Another consideration is the financial cost associated with this treatment. The Da Vinci system, including software upgrades, must be balanced against the potentially shorter hospital lengths of stay and reduced hospital costs.^8^

Few previous studies have evaluated the functional or quality of life outcomes following TORS for OPSCC compared to other treatments, including RT with or without concurrent chemotherapy. The findings of these studies have been ambivalent. Lee et al found improved MD Anderson Dysphagia Inventory (MDADI) scores with TORS at 12 months relative to open mandibulotomy.^9^ Previous reviews have also suggested TORS could be superior to RT in preserving swallowing^10^, ^11^ although these reviews have mostly included retrospective, non‐randomized studies with relatively short follow‐up times. A review by Campo et al reported no statistically significant difference in the mean MDADI scores following TORS or RT for OPSCC at 12 months.^12^ On the other hand, the first randomized study on the subject, the ORATOR trial, found superior swallowing‐related quality of life outcomes among patients receiving RT treatment, measured by MDADI scores after 1 year of follow‐up.^13^

As such, this systematic review aims to compare a wide variety of outcomes amongst patients diagnosed with OPSCC who were treated with TORS against those who were treated with (chemo)radiotherapy (CRT). Several key outcomes are evaluated, including pain scores and the impact on swallowing and speaking function.

## Methodology

The systematic review was reported in accordance with the Preferred Reporting Items for Systematic reviews and Meta‐analyses (PRISMA) guidelines.^14^ The review protocol was registered with the International Prospective Register of Systematic Reviews (PROSPERO) on September 20, 2023, and is publicly available (registration number CRD42023464144).^15^ The full protocol is included in Supplemental Section S1, available online.

### Search

We searched the following databases from 1997 to September 25, 2023: Medline (1997‐present), EMBASE (1997‐present), Web of Science Core Collection (1997‐present), and the Cochrane Library (1997‐present). The search strategy involves first identifying terms related to cancer, tumors, or oncology, using keywords such as “(cancer* or carcinoma* or neoplasm* or malignan* or tumor* or tumour* or oncolog* or sarcoma* or metasta* or lesion*)” or terms related to abnormal or pathological growths: “(abnormal or pathological adj (growth* or mass*)).” This is then filtered by terms specific to head and neck regions, including “(craniofacial or facial or head or neck or larynx or pharynx or nasal or skull or jaw or salivary glands or tongue or esophag*).” Finally, the search includes terms related to robotic or computer‐assisted surgery, such as “((robot* or mechan* or automat* or technology assist* or computer guided) adj3 (surg* or procedure*)).”

The authors chose to include studies published after 1997, as the United States Food & Drug Administration cleared Intuitive Surgical's da Vinci surgical system for assisting in surgeries in that year. The search methodology used is attached in Supporting Information, available online.^16^

Gray literature was searched on the World Health Organization International Clinical Trials Registry Platform, ScanMedicine, UK Clinical Trials Gateway, ClinicalTrials.gov, EThOS, and Google Advanced Search.

The whole search record was saved in EndNote^17^ and screened by at least two authors on Rayyan.^18^

### Eligibility Criteria

Studies were considered eligible if they met the following inclusion criteria:

Inclusion criteria:

1. Studies must be one of randomized control studies, prospective studies, case‐controlled and cohort studies.
2. Patients enrolled in the study must be aged 18 or above.
3. The study involves TORS with or without adjuvant therapy and compares outcomes against CRT or RT alone.
4. The study assesses the swallowing and speaking functions, the incidence of tracheostomies, the use of nasogastric tube, the use of gastrostomy tube, recovery metrics or pain levels.
5. The setting of the study is in a hospital.
6. The study must be written in English, Spanish, or Chinese.

Exclusion criteria:

1. Studies cannot be presented as abstract‐only papers, preceding papers, conference papers, editorials, theses, reviews, and books.

### Study Selection

Titles and abstracts were reviewed by two independent reviewers against the inclusion criteria, and discrepancies were resolved through discussion with a third independent reviewer. All papers were double‐screened. Any discrepancies that arose were identified, addressed, and subjected to group discussions.

Following title and abstract screening, full texts were assessed against pre‐defined inclusion criteria. Cross‐referencing of included papers was performed to identify any previously overlooked articles.

Following this, a “snowballing” technique was applied, wherein all articles citing the included papers underwent screening of their title and abstract. Any newly discovered articles were then subjected to another round of title, abstract, and full‐text screening, if deemed relevant and appropriate.

### Data Extraction and Risk‐of‐Bias Assessment

Relevant data were extracted from each included study, including study characteristics, baseline demographic and clinical information (such as tumor stages and nodal stages), and outcomes for individuals in both the TORS and CRT groups. Data extraction was carried out by two independent reviewers.

Three independent reviewers conducted critical appraisals using the Cochrane Risk of Bias 2 (RoB 2) tool^19^ for the randomized controlled trial, and the Risk of Bias in Non‐randomized Studies of Interventions (ROBINS‐I) tool for the non‐randomized study.^20^ Studies exhibiting a critical risk of bias would be excluded from the analysis.

### Statistical Analysis

Following data extraction, Student's *t* test assuming equal variance was used to determine if there are any significant differences in patient selection based on tumor and nodal staging. Analyses were performed using Microsoft Excel v16.78.^21^

A meta‐analysis was conducted to evaluate pooled effects at 3, 6, and 12 months. We extracted or calculated log‐transformed odds ratios (ORs) and standard errors. Where data were missing, confidence intervals (CIs) were inferred or imputed. Pooled estimates were computed using fixed and random‐effects models. Heterogeneity was assessed using *I*², *τ*², and Cochran's *Q* test. Study weights were assigned via inverse‐variance. A significance level of *P* < .05 was used for statistical tests. Analyses were performed using R version 4.4.0^22^ with the meta package version 8.0‐1.^23^ Forest and funnel plots, if applicable, are generated to visualize individual study estimates and pooled ORs at each time point. GRADE methodology was used to evaluate evidence certainty.^24^

### Deviations From the Protocol

HPV status and the presence of the p16 intracellular protein, involved in cell cycle checkpoint and retinoblastoma control, were used in the final data extraction and analysis. In addition, TORS was compared with chemotherapy and RT, the current conventional treatments, to more effectively address the review question. Consequently, factors such as the length of hospital stay and recovery time were excluded given the inherent differences between surgical and medical interventions making it inappropriate to compare such parameters in this context.

## Results

### Paper Inclusion

**Figure 1:**
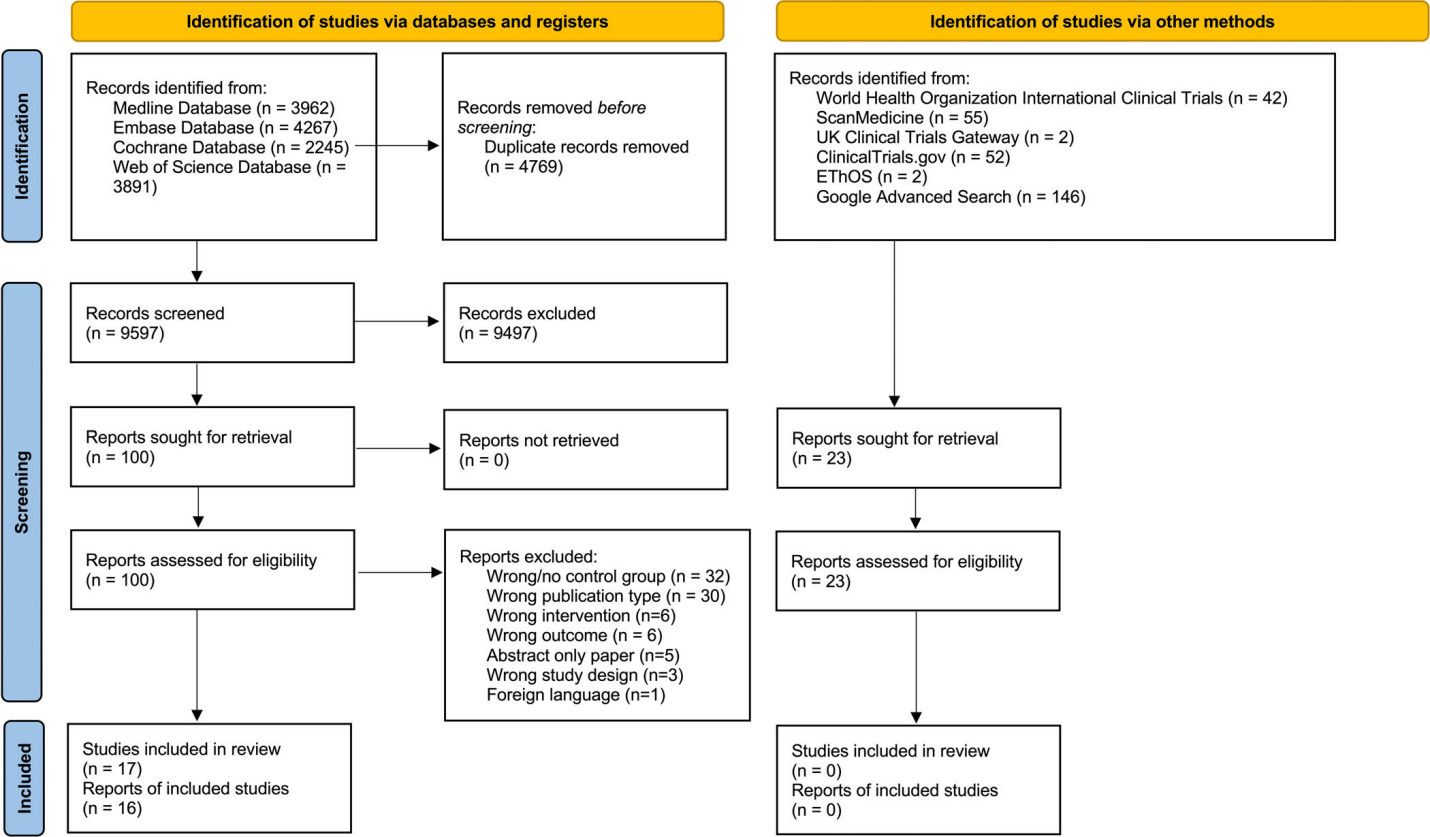
Preferred Reporting Items for Systematic reviews and Meta‐analyses (PRISMA) flow diagram of study selection. In total, 10,204 records were identified, 100 assessed for eligibility, and 17 studies included. Exclusions due to no control group, wrong design/outcome, publication type, abstract‐only, or language.

Following full‐text screenings, 17 papers met the inclusion criteria. Two papers presented findings derived from an identical cohort of patients,^13^, ^25^ thus only the primary analysis was included.^13^ As a result, 16 studies are included in the final analysis (Figure 1). While Hutcheson et al (2019)^26^ and Barbon et al (2021)^27^ studied the same group of patients, they were both included as they presented a distinct set of patient‐reported outcomes.

### Risk‐of‐Bias Assessment

Cochrane's RoB‐2 tool^19^ was used to assess the only randomized‐control study included^13^ revealing some concerns for bias.

The ROBINS‐I tool was applied to non‐randomized studies included in the analysis.^20^ Among the identified studies, three were classified as having a low risk of bias,^27^, ^28^, ^29^ eight as moderate,^13^, ^26^, ^30^, ^31^, ^32^, ^33^, ^34^, ^35^ five as serious,^36^, ^37^, ^38^, ^39^, ^40^ and none as critical. This is illustrated by the Robvis risk‐of‐bias plots,^41^ with detailed breakdowns in Supplemental Figures S1 and S3, available online, for the ROBINS‐I and RoB‐2 tools, respectively, in Supplemental Section S2, available online.

Table 1 summarizes key outcomes, including MDADI scores and gastrostomy tube dependence rates over time, along with relative and absolute effects and GRADE certainty ratings. Full details on study characteristics, statistical methods, and GRADE assessments are provided in Supplemental Section S3, available online.

**Table 1:** GRADE Summary Comparing Transoral Robotic Surgery (TORS) Versus Non‐TORS for Swallowing and Gastrostomy Outcomes

Outcome	No. of studies	Study design	Relative effect (95% CI)	Absolute effect	Certainty (•)	Notes (downgrades/upgrades)
Swallowing function (MDADI) at 3 to 6/6 mo	3	Design type: 3 observational; orientation: 3 prospective	TORS: SMD −0.61 (approx. −0.79 to −0.42) Control: SMD −0.86 (approx. −1.01 to −0.72)	TORS: −7.05 points Control: −11.02 points	**•••**◯ Moderate	Lower MDADI scores reflect worse swallowing function. Control group showed a greater decline, suggesting TORS may better preserve function
Swallowing function (MDADI) at 12 mo	3	Design type: 2 observational, 1 randomized controlled trial; orientation: 3 prospective	TORS: SMD –0.63 (95% CI: –1.21 to –0.06) Control: SMD –1.68 (95% CI: –2.79 to –0.57)	TORS: –3.2 to –12.8 points Control: –5.8 to –18 points	**•••**◯ Moderate	Downgraded for risk of bias and imprecision in some studies. Control group consistently showed greater decline. TORS may better preserve swallowing function
Gastrostomy tubes (at 3 mo)	2	Design type: 3 observational; orientation: 2 retrospective	0.42 [0.03‐5.16] (random‐effects)	↓ ~22 fewer events per 100	**••**◯◯ Low	Downgraded: inconsistency (substantial heterogeneity), imprecision (one study had wide CI and no significance; limited sample size)
Gastrostomy tubes (at 6 mo)	4	Design type: 4 observational; orientation: 1 prospective, 3 retrospective	0.14 [0.017‐1.17] (random‐effects)	↓ ~30 fewer events per 100	**••**◯◯ Low	Downgraded: inconsistency (substantial heterogeneity), imprecision (very wide CI)
Gastrostomy tubes (at 12 mo)	7	Design type: 5 observational, 1 case‐control, 1 randomized controlled trial; orientation: 3 prospective, 4 retrospective	0.35 [0.17‐0.73] (random‐effects)	↓ ~12‐20 fewer events per 100	**••**◯◯ Low	Downgraded: inconsistency (substantial heterogeneity), imprecision (very wide CI)
Pain level (EORTC H&N 35 at 12 mo)	2	Design type: 1 observational, 1 randomized controlled trial; orientation: 2 prospective	Inconsistent (−4.3 to +2.2)	Inconsistent differences	**••**◯◯ Low	Downgraded: inconsistency and imprecision due to unknown sample sizes and opposing results

Abbreviations: EORTC H&N, European Organisation for Research and Treatment of Cancer Head & Neck; MDADI, MD Anderson Dysphagia Inventory, SMD, standardized mean difference.

### Demographics

Demographic data from each study are presented in Table 2. The included studies comprised 2185 patients. A total of 1054 patients underwent TORS as part of the treatment, whereas 1131 patients received primary CRT, regardless of the HPV status. None of the included studies found a significant difference in the male sex distribution (*P* = .43) or mean age (*P* = .90) between the TORS and CRT cohorts.

**Table 2:** Demographic and Treatment Characteristics of Patients in Transoral Robotic Surgery (TORS) Studies

	TORS					Adjuvant therapies		Control treatment					
Author	Number of patients	% female	% male	Age (mean)	Age (median)	Type	Number of patients (%)	Control treatment	Number of patients	% female	% male	Age (mean)	Age (median)
Amin et al^31^	363	22.9	77.1	69.2	n.r.	None	‐	Radiotherapy or chemoradiotherapy	363	22.9	76.9	68.6	n.r.
Barbon et al^27^	75	86.7	13.3	58	n.r.	CRT	15 (20%)	Radiotherapy	182	13.7	86.3	59	n.r.
Hutcheson et al^26^													
Barbon et al^34^	38	15.8	84.2	n.r.	58	CRT, RT	8 (21%), 9 (24%)	Radiotherapy	97	18.6	81.4	n.r.	57.5
Chen et al^30^	31	16.1	83.9	n.r.	52	CRT, RT	5 (16%), 26 (84%)	Chemoradiotherapy	31	16.1	83.9	n.r.	53
Dhanireddy et al^29^	65	26.2	73.8	n.r.	61	CRT, RT	24 (37%), 24 (37%)	Chemoradiation	54	53.7	46.3	n.r.	57.9
Genden et al^39^	30	13.3	86.7	n.r.	52	CRT, RT	11 (37%), 14 (47%)	Chemoradiation	26	23.1	76.9	n.r.	58
Hughes et al^33^	116	n.r.	n.r.	n.r.	59	CRT, RT	33 (28%), 30 (26%)	Radiotherapy	51	n.r.	n.r.	n.r.	58
Kaffenberger et al^37^	29	13.8	86.2	56.7	n.r.	CRT, RT	20 (27%), 9 (12%)	Chemoradiation or radiation	44	15.9	84.1	57.6	n.r.
Ling et al^28^	92	16.3	83.7	n.r.	56	CRT, RT	37 (40%), 15 (16%)	Chemoradiotherapy	46	17.4	82.6	n.r.	58
Meccariello et al^38^	60	21.7	78.3	64.3	n.r.	CRT, RT	20 (33%), 19 (32%)	Chemoradiotherapy	69	20.3	79.7	61.1	n.r.
More et al^32^	20	40	60	n.r.	54	CRT, RT	12 (60%), 8 (40%)	Chemoradiotherapy	20	30	70	n.r.	56
Nichols et al^13^	34	17.6	82.4	n.r.	58.1	CRT, RT	8 (24%), 16 (47%)	Radiotherapy	34	8.8	91.2	n.r.	60
Scott et al^36^	31	29	71	n.r.	59	CRT	4 (13%)	Radiotherapy ± chemotherapy	13	15.4	84.6	n.r.	58
Scott et al^40^	31	29	71	n.r.	59	CRT	6 (19%)	Radiotherapy	13	15.4	84.6	n.r.	58
Sharma et al^35^	39	5.1	94.8	n.r.	58	CRT, RT	11 (28%), 24 (62%)	Radiotherapy/chemoradiotherapy	88	14	86	n.r.	57
Average	70	25.3	74.7	62.1	56.9				75	20.4	79.6	61.6	57.3

Abbreviations: CRT, (chemo)radiotherapy; n.r., not reported; RT, radiotherapy.

### Cancer Stage

Statistical testing using *t* test revealed no significant differences in the distribution of tumor stage (*P* = .85) and nodal stage (*P* = .89) between the TORS and CRT groups. Six studies matched the TORS and CRT cohorts by T stage,^13^, ^29^, ^30^, ^32^, ^35^, ^38^ while four were matched by N stage.^13^, ^30^, ^38^, ^39^ Three studies identified a significant difference in tumor staging between the TORS and CRT groups,^33^, ^34^, ^39^ and five studies observed a significant difference in node staging between the two groups.^27^, ^28^, ^29^, ^33^, ^34^ Amin et al (2023) studied the differences in functional outcomes between patients receiving surgery and those receiving CRT for the treatment of T1‐T2 OPSCC. However, the study did not specify the population by tumor or nodal grading.^31^ In all instances, the TORS group had a lower tumor and nodal staging. Table 3 presents patient demographics, cancer stages, and outcomes by treatment type, specifically TORS and CRT. Table 4 compares the usage of adjuvant treatments in TORS and control groups, detailing indications, treatment types, and patient distribution.

**Table 3:** Tumor and Nodal Staging Characteristics in Transoral Robotic Surgery (TORS) Cohorts

					TORS with or without adjuvant therapy												Control treatment														
Author	Subsite	Cohort matching for T stage	Cohort matching for N stage	Cancer staging standards	T0	T1	T2	T3	T4	Tx	N0	N1	N2	N3	N4	Not reported	T0	T1	T2	T3	T4	Tx	N0	N1	N2	N3	N4	Not reported	*P*‐value (tumor)	*P*‐value (node)	Unilateral or bilateral node status
Chen et al^30^	Oropharynx	Matched	Matched	AJCC	0	14	12	5	0	0	0	5	26	0	0	0	0	14	12	5	0	0	0	5	26	0	0	0	n.r.	n.r.	Implied from N staging that some bilateral node status patients included
Amin et al^31^	Oropharynx	Not matched	Not matched	n.r.	0	0	0	0	0	363	0	0	0	0	0	363	0	0	0	0	0	363	0	0	0	0	0	363	n.r.	n.r.	n.r.
Scott et al^40^	Oropharynx	Not matched	Not matched	AJCC 7th edition	0	15	16	0	0	0	16	15	0	0	0	0	0	6	4	1	2	0	0	12	1	0	0	0	n.r.	n.r.	Not reported
Barbon et al^27^/Hutcheson et al^26^	Oropharynx	Not matched	Not matched	AJCC 7th edition	0	40	34	1	0	0	31	15	29	0	0	0	0	84	92	6	0	0	19	20	143	0	0	0	.5	<.001	All unilateral node status (implied from N stage data)
More et al^32^	Tonsil, base of tongue, oropharynx, epiglottis, tonsil	Matched	Not matched	AJCC	0	6	8	6	0	0	1	8	11	0	0	0	0	6	6	8	0	0	0	8	12	0	0	0	.92	>.99	n.r.
Scott et al^36^	Oropharynx	Not matched	Not matched	Union for International Cancer Control TNM Atlas 7th edition	0	15	16	0	0	0	16	15	0	0	0	0	0	6	4	1	2	0	0	12	1	0	0	0	n.r.	n.r.	n.r.
Ling et al^28^	Oropharynx	Not matched	Not matched	n.r.	4	53	35	0	0	0	19	27	46	0	0	0	1	17	28	0	0	0	1	10	35	0	0	0	.08	<.001	n.r.
Dhanireddy et al^29^	Oropharynx	Matched	Not matched	AJCC 7th edition	0	20	45	0	0	0	14	13	37	1	0	0	0	22	32	0	0	0	6	2	43	3	0	0	.257	<.001	Implied from N staging that some bilateral node status patients included
Kaffenberger et al^37^	Oropharynx	Not matched	Not matched	AJCC 7th edition	0	16	10	3	0	0	1	3	22	0	0	0	0	17	15	4	8	0	0	6	36	2	0	0	.074	.442	Implied from N staging that some bilateral node status patients included
Nichols et al^13^	Oropharynx	Matched	Matched	AJCC 7th edition	0	17	17	0	0	0	9	7	18	0	0	0	0	13	21	0	0	0	12	5	17	0	0	0	n.r.	n.r.	n.r.
Sharma et al^35^	Oropharynx	Matched	Not matched	n.r.	0	16	18	6	0	0	5	7	24	3	0	0	0	22	51	15	0	0	7	8	64	9	0	0	n.r.	n.r.	Implied from N staging that some bilateral node status patients included
Hughes et al^33^	Oropharynx	Not matched	Not matched	AJCC 8th edition	3	60	53	0	0	0	25	84	7	0	0	0	0	10	41	0	0	0	1	33	17	0	0	0	<.001	<.001	All unilateral node status (implied from N stage data)
Barbon et al^34^	Tonsil	Not matched	Not matched	AJCC 7th edition	0	19	18	1	0	0	16	22	0	0	0	0	0	56	77	3	0	0	10	87	0	0	0	0	<.001	<.001	No bilateral node status patients included
Meccariello et al^38^	Oropharynx	Matched	Matched	AJCC 8th edition	3	27	22	8	0	0	8	15	30	7	0	0	2	10	21	17	18	0	3	41	21	3	0	0	n.r.	n.r.	Implied from N staging that some bilateral node status patients included
Genden et al^39^	Base of tongue, tonsil, oropharyngeal wall, soft palate, retromolar trigone, larynx, hypopharynx, nasopharynx, unknown primary	Not matched	Matched	AJCC	0	14	16	0	0	0	6	10	14	0	0	0	1	4	14	3	4	0	4	7	15	0	0	0	.003	.18	Implied from N staging that some bilateral node status patients included
Sum					10	372	354	31	0	363	198	261	293	11	0	363	4	371	510	69	34	363	82	276	574	17	0	363			

Abbreviation: AJCC, American Joint Committee on Cancer; n.r., not reported.

**Table 4:** Adjuvant Therapy Use and Indications in Transoral Robotic Surgery (TORS) and Control Groups

	TORS with or without adjuvant therapy			Control treatment			
	Adjuvant	Adjuvant			Adjuvant		
Author	Indication	Type	Number of patients (%)	Control treatment	Indication	Adjuvant type	Number of patients (%)
Amin et al^31^	‐	None	‐	RT/CRT	‐	None	‐
Barbon et al^27^	n.r.	CRT	15 (20%)	RT	n.r.	CRT	152 (84%)
Hutcheson et al^26^		RT	22 (29.3%)				
Barbon et al^34^	n.r.	CRT	8 (21%)	Unilateral RT	Chemotherapy:	Chemotherapy	28 (76%),
		RT	9 (24%)	Bilateral RT	Stage T3/T4	Chemotherapy	46 (77%)
Chen et al^30^	n.r.	CRT	5 (16%)	CRT	‐	None	‐
		RT	26 (84%)				
Dhanireddy et al^29^	CRT:	CRT	24 (37%)	CRT	‐	None	‐
	Extracapsular extension, the presence of extensive (>5) nodal disease						
	RT:	RT	24 (37%)				
	Close margins (<2 mm) perineural or lymphovascular invasion, N2 (a or b) disease						
Genden et al^39^	CRT:	CRT	11 (37%)	CRT	‐	None	‐
	Pathologic ENE, positive margins	RT	14 (47%)				
	RT: N2b/N2c/N3 disease, close final margins, T3 tumors						
Hughes et al^33^	CRT:	CRT	33 (28%)	RT	Chemotherapy:	CRT	50 (98.0%)
	Positive margin or ENE				Positive margin or ENE		
	RT:	RT	30 (26%)				
	pT3 or higher tumor, pN2 or greater, perineural invasion, lymphovascular invasion, close margin (<2 mm)						
Kaffenberger et al^37^	n.r	CRT	20 (27%)	CRT	‐	None	‐
		RT	9 (12%)				
Ling et al^28^	CRT: extracapsular extension or positive margins	CRT,	37 (40%)	CRT	‐	None	‐
	RT: ⩾N2 disease, angiolymphatic or perineural invasion, and close (<3 mm) margins	RT	15 (16%)				
Meccariello et al^38^	CRT:	CRT	20 (33%)	CRT	‐	None	‐
	Pathologic ENE or positive margins						
	RT:	RT	19 (32%)				
	N2b/N2c/N3 disease, close final margins, and all patients with T3 tumors						
More et al^32^	CRT:	CRT	12 (60%)				
	Extracapsular spread in cervical nodes, with surgical margins <10 mm			CRT	‐	None	‐
	RT:	RT	8 (40%)				
	T3 primary tumor or N2 disease						
Nichols et al^13^	CRT:	CRT	8 (24%)	RT	N1/2	Chemotherapy	23 (68%)
	Positive margins or ENE						
	RT:	RT	16 (47%)				
	pT3 or pT4 disease, close resection margins [<2 mm], nodal disease, or lymphovascular invasion						
Scott et al^36^	CRT:	CRT	5 (16%)	RT	Chemotherapy:		
	Positive margins, ENE, or more than one involved lymph node				Positive margins, ENE, or more than one involved lymph node	Chemotherapy	9 (69%)
Scott et al^40^	CRT:				Chemotherapy:		
	Positive margins (≥2 mm), ENE, or spread to more than one lymph node	CRT	6 (19%)	RT	Positive margins (≥2 mm), ENE	Chemotherapy	9 (69%)
Sharma et al^35^	n.r.	CRT	11 (28%)	CRT	‐	None	‐
		RT	24 (62%)	RT			

Abbreviations: CRT, (chemo)radiotherapy; ENE, extranodal extension; n.r., not reported; RT, radiotherapy.

Based on the reported statistics, patients with positive HPV‐associated status are more likely to be observed than those with negative status, despite a high number of unknown or undetermined HPV‐associated statuses in four studies.^28^, ^29^, ^35^, ^38^ Two studies performed stage‐matching with respect to HPV‐associated status.^13^, ^30^ Two studies included patients based on HPV status without specifying specific strains.^27^, ^34^ Four studies did not report any data on HPV‐associated status.^31^, ^32^, ^36^, ^39^ No study performed a stratified analysis based on HPV‐associated status in relation to functional outcomes. Table 5 summarizes the status of HPV and its associated strains among patients in the included studies. It provides an overview of HPV prevalence and strain distribution across the patient cohort.

**Table 5:** Human Papillomavirus (HPV) Status in Transoral Robotic Surgery (TORS) and Control Cohorts

	TORS with or without adjuvant therapy			Control treatment		
Author	Positive	Negative	Not determined/unknown	Positive	Negative	Not determined/unknown
Amin et al^31^	n.r.					
Barbon et al^27^/Hutcheson et al^26^	All had HPV‐associated disease but p16 n.r.					
Barbon et al^34^	All had HPV‐associated disease but p16 n.r.					
Chen et al^30^	20 (65%)	11 (35%)	0 (0%)	20 (65%)	11 (35%)	0 (0%)
Dhanireddy et al^29^	52 (80%)	10 (15%)	3 (5%)	25 (46%)	3 (6%)	26 (48%)
Genden et al^39^	n.r.					
Hughes et al^33^	All had either p16+ or HPV PCR					
Kaffenberger et al^37^	29 (100%)	0 (0%)	0 (0%)	37 (84%)	3 (7%)	4 (9%)
Ling et al^28^	TORS alone: 31 (77.5%)	TORS alone: 8 (20%)	TORS alone: 1 (2.5%)	Definitive CRT: 19 (41.3%)	Definitive CRT: 4 (8.7%)	Definitive CRT: 23 (50.0%)
	TORS + adjuvant RT or CRT: 42 (80.8%)	TORS + adjuvant RT or CRT: 6 (1.9%)	TORS + adjuvant RT or CRT: 4 (7.7%)			
Meccariello et al^38^	35 (58.3%)	19 (31.7%)	6 (10%)	42 (60.9%)	6 (8.7%)	21 (30.4%)
More et al^32^	n.r.					
Nichols et al^13^	30 (88%)	4 (12%)	0 (0%)	30 (88%)	4 (12%)	0 (0%)
Scott et al^36^	n.r.					
Scott et al^40^	24 (77%)	n.r.	n.r.	12 (92.3%)	n.r.	
Sharma et al^35^	30 (76.9%)	1 (2.6%)	8 (20.5%)	30 (34.1%)	7 (8.0%)	51 (58.0%)

Abbreviations: CRT, (chemo)radiotherapy; n.r., not reported; RT, radiotherapy.

### Swallowing Functions

**Figure 2:**
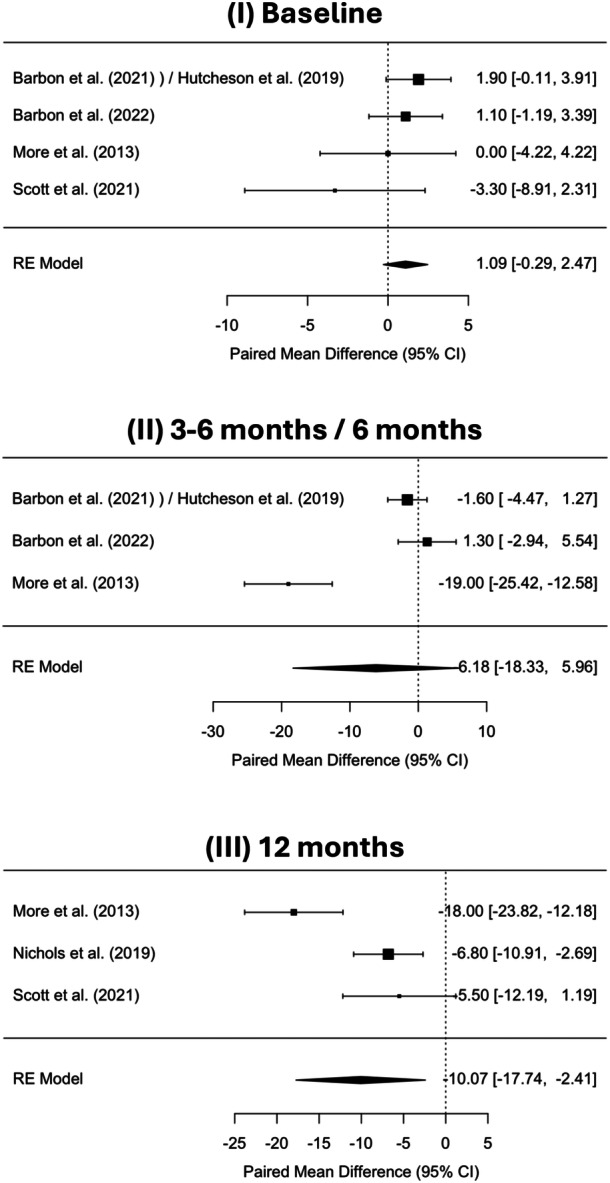
Forest plots of MD Anderson Dysphagia Inventory (MDADI) swallowing scores comparing transoral robotic surgery (TORS) and (chemo)radiotherapy (CRT) at baseline, 3 to 6 months, and 12 months. Squares show study estimates/weights; diamonds pooled random‐effects results (95% CI).

For patient‐reported swallowing assessments, five studies used the MDADI scoring tool.^13^, ^27^, ^32^, ^34^, ^36^ Scott et al (2021) reported no significant changes in overall MDADI scores with both TORS and RT treatment group at 12 months, despite some significant changes in individual question scores in post hoc analysis.^36^ However, Nichols et al (2019) found that TORS was worse than the RT group (80.1 vs 86.9, *P* = .042) over the same period.^13^ More et al (2013) identified statistically significant improvements in MDADI scores in the TORS group at 6‐ and 12‐month post‐procedure, compared to CRT but no significant difference at 3‐month.^32^ Barbon et al (2021) overall found no statistically significant differences in MDADI scores between TORS and the RT group at 3 or 6 months.^27^ Pooled analysis showed no significant difference in MDADI swallowing scores between TORS and CRT at baseline (95% CI = –0.29 to 2.47) and at 3 to 6 months (95% CI = −18.33 to 5.96) (Figure 2). The wide CI suggests substantial variability between studies, without favoring either arm of treatment. However, the pooled estimate at 12 months was below zero (95% CI –17.74 to –2.41), indicating a statistically significant difference in favor of CRT (Figure 2).

Various swallowing assessments were used, as outlined in Table 6, with detailed findings in Supplemental Section S4, available online. Dynamic Imaging Grade of Swallowing Toxicity (DIGEST) was performed in four studies.^27^, ^34^, ^36^, ^40^ DIGEST scores were assessed at multiple time points, but none of the studies described a significant difference in score between the TORS and CRT groups.^27^, ^34^, ^36^, ^40^

**Table 6:** Swallowing Function Outcomes in Transoral Robotic Surgery (TORS) Versus Control Groups

Author	Time points, mo	Scoring metric	TORS group score	Control group score	*P*‐value
Barbon et al^34^	0; 3‐6; 24	MDADI	92; 83; 85.5	92.9/93.2; 88.0/82.0; 86.4/n.r. (unilateral RT/bilateral RT)	.9; .38; .99
Nichols et al^13^	12	MDADI	80.1	86.9	.04^a^
Scott et al^36^	0; 12	MDADI	93.3; 90.5	90; 85	n.r.; n.r.
More et al^32^	3; 6; 12	MDADI	62; 76; 78	56; 57; 60	Insignificant; .004^a^; .006^a^
Barbon et al^27^	0; 3‐6	DIGEST grade ≥1 (%)	25%; 45%	16%; 42%	.06; .93
Barbon et al^27^	3‐6	MBSImP ‐ laryngeal vestibule closure (%)	27%	41%	.02^a^
Barbon et al^27^	3‐6	MBSImP ‐ pharyngeal contraction (%)	62%	53%	.001^a^
Scott et al^36^	3; 12	DIGEST grade 1 or 2 (%)	54.8%; 29.0%	23.1%; 45.5%	n.r.; n.r.
Scott et al^40^	12; 36	DIGEST grade 1 or 2 (%)	29%; 11.5%	45.5%; 9.09%	n.r.; n.r.
Genden et al^39^	0.5^b^	FOIS	5.5 ± 0.2	3.3 ± 0.6	<.001^a^
Hughes et al^33^	12 mo	FOIS change distribution	−6: 1.0; −5: 1.0; −4: 1.0; −3: 1.0; −2: 2.9; −1: 29.4; 0: 58.8; +1: 4.9; +2: 0	−6: 1.0; −5: 1.0; −4: 2.0; −3: 2.0; −2: 10.0; −1: 24.0; 0: 34.8; +1: 19.2; +2: 2.2	.008^a^

Abbreviations: DIGEST, Dynamic Imaging Grade of Swallowing Toxicity; FOIS, Functional Oral Intake Scale; MBSImP, Modified Barium Swallow Impairment Profile; MDADI, MD Anderson Dysphagia Inventory; n.r., not reported; RT, radiotherapy.

^a^ Statistically significant.

^b^ No outcome data were reported for later time points (3‐12 months), though the authors stated results had no significant difference.

A study described swallowing function using the Modified Barium Swallow Impairment Profile (MBSImP), they found significantly more pharyngeal contraction impairment for TORS but reduced laryngeal vestibular closure impairment compared with their control group who had been treated with RT.^27^ Of the two studies that used the Functional Oral Intake Scale (FOIS), one reported less deterioration in TORS patients compared to those receiving CRT after 1 year,^33^ while the other found no significant difference between the groups.^39^

Quality of life measures is summarized in Table 7. A study employed the University of Washington Quality of Life questionnaire (UW‐QOL) to assess patient outcomes.^28^ The study revealed that patients undergoing TORS consistently and significantly scored higher in the saliva domain of quality‐of‐life assessments at 1‐, 6‐, 12‐, and 24‐month posttreatment compared to those receiving CRT (*P* < .001, *P* = .025, *P* = .017, and *P* = .011).^28^

**Table 7:** Quality of Life Outcomes Following Transoral Robotic Surgery (TORS) Versus Control Groups

Author	Metric	Timescale	TORS ± adjuvant therapy	Control treatment	*P*‐value
Ling et al^28^	UW‐QOL version 4	1 mo	52 (27)	64 (24)	n.r.
		6 mo	81 (22)	83 (21)	n.r.
		12 mo	89 (23)	78 (27)	n.r.
		24 mo	86 (21)	82 (25)	n.r.
Nichols et al^13^	EORTC QLQ‐C30	1 y	21.8 (25.2)	8.0 (16.3)	.018
	EORTC H&N 35	1 y	13.3 (14.9)	9.0 (12.4)	.23
Scott et al^36^	EORTC H&N 35	Baseline	9.1 (11.7)	29.9 (19.9)	n.r.
		12 mo	8.9 (13.8)	11.1 (14.8)	n.r.
		Overall change	Mean change −0.3	Mean change −16.8	n.r.
			(*P* = .919)	(*P* = .037)	

Abbreviations: EORTC H&N 35, European Organisation for Research and Treatment of Cancer Head & Neck 35; EORTC QLQ‐C30, European Organisation for Research and Treatment of Cancer Core Quality of Life questionnaire; n.r., not reported; UW‐QOL, University of Washington Quality of Life questionnaire.

Scott et al (2023) used European Organisation for Research and Treatment of Cancer Core Quality of Life questionnaire (EORTC QLQ‐C30) and Head & Neck 35 (H&N 35); however, no statistically meaningful difference was elicited.^40^ The EORTC QLQ‐C30 was further used in Scott et al (2021), which exhibited favorable outcomes for global quality of life for TORS (*P* = .004) and RT (*P* = .032).^36^ There were more favorable outcomes for emotion in TORS (*P* > .001) than RT (*P* = .009); and for appetite for TORS (*P* = .032) than RT (*P* = .674).^36^ Kaffenberger et al (2021) in their study utilized the Eating Assessment Tool‐10 (EAT‐10) and reported no statistically significant differences over a median follow‐up duration of 29.7 months between the TORS and control groups (*P* = .18), for which the control group received CRT or RT.^37^

### Gastrostomy Tubes and Tracheostomies

Eight studies described gastrostomy tube insertion^13^, ^29^, ^30^, ^31^, ^32^, ^33^, ^35^, ^39^ at various timepoints. Usage rates and comparative data are summarized in Table 8. One study reported significantly lower gastrostomy tube rates in the TORS group at 3 months, three out of four studies at 6 months, and two out of five at 12 months.

**Table 8:** Gastrostomy Tube Dependence Following Transoral Robotic Surgery (TORS) Versus Control Treatment

		TORS ± adjuvant therapy		Control treatment			
Author	Time scale	Number	Percentage (%)	Number	Percentage (%)	Odd ratio	Significance
Sharma et al^35^	At 3 mo	3	9	37	45	0.115	<.001
Amin et al^31^	At 6 mo	n.r.	n.r.	n.r.	n.r.	0.455	.23
Dhanireddy et al^29^	At 6 mo	n.r.	n.r.	n.r.	n.r.	1.84	.31
More et al^32^	At 6 mo	0	0	12	60	0	<.001
Sharma et al^35^	At 6 mo	1	0	18	72	0	.04
Amin et al^31^	At 12 mo	n.r.	n.r.	n.r.	n.r.	0.846	.71
Chen et al^30^	At 12 mo	1	3.23	3	9.68	1.30	.01
Genden et al^39^	At 12 mo	0	0	1	3.85	0.00	.49
Hughes et al^33^	At 12 mo	4	3.5	12	23.5	1.34	<.001
More et al^32^	At 12 mo	0	0	1	5	0.00	1.00
Nichols et al^13^	At 12 mo	0	0	1	2.94	0.00	1.00
Sharma et al^35^	At 12 mo	1	3	7	11	1.36	.15

Abbreviation: n.r., not reported.

**Figure 3:**
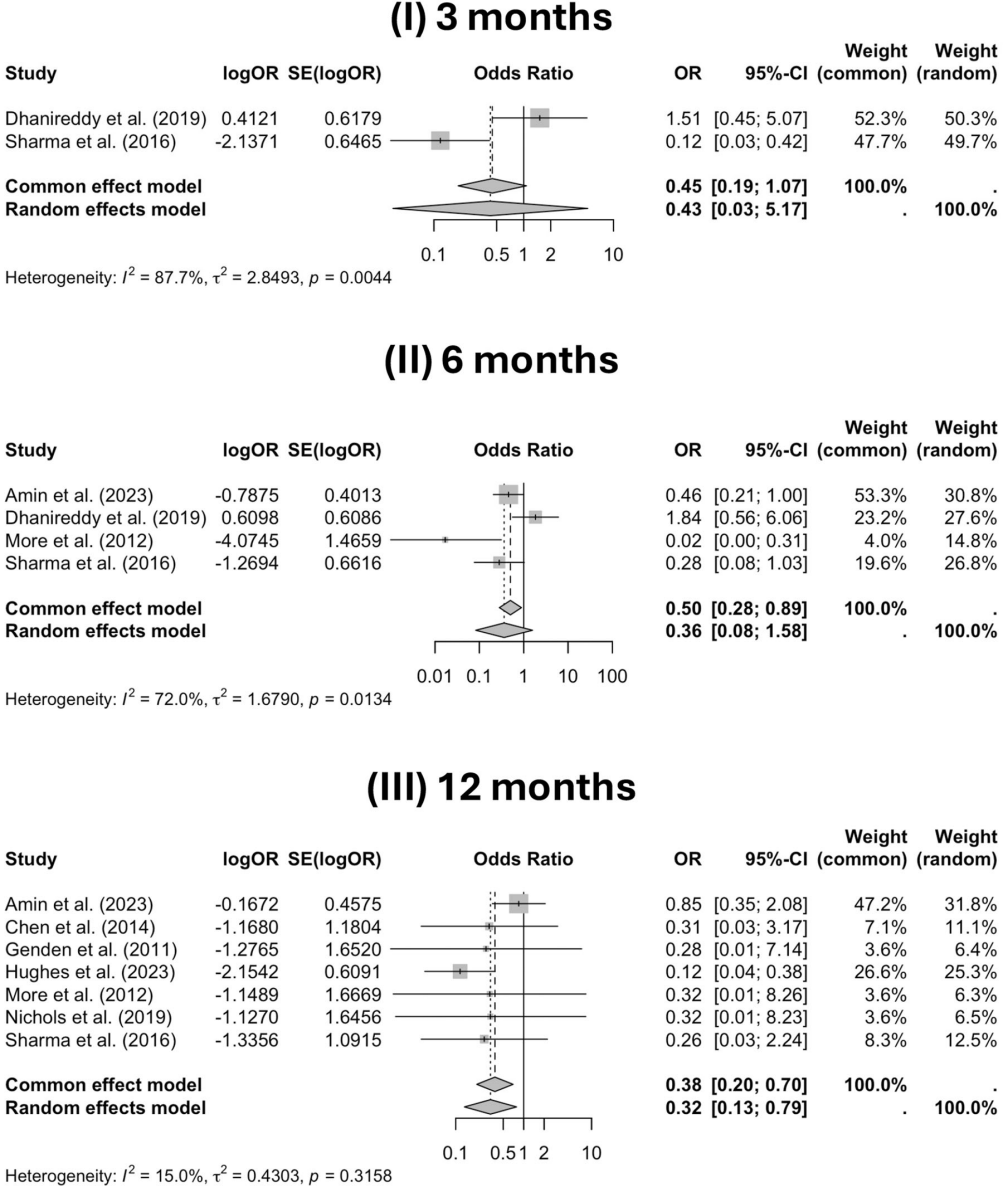
Forest plots of gastrostomy tube dependence at 3, 6, and 12 months, comparing transoral robotic surgery (TORS) versus controls. Includes study estimates, pooled random‐effects results, and heterogeneity statistics. OR, odds ratio.

The meta‐analysis showed high heterogeneity at 3 and 6 months, which substantially decreased by 12 months (Figure 3). At 3 months, *I*² was 87.7%, indicating most variation was due to true differences between studies. At 6 months, *I*² was 72.0% with a high *τ*² and a significant *P*‐value (<.05), confirming substantial heterogeneity. By 12 months, heterogeneity was low. The random‐effects model demonstrated no significant differences in gastrostomy tube dependence between groups at 3 and 6 months; however, a statistically significant reduction was observed in the TORS group at 12 months.

One of the studies included reported the requirement for tracheostomy as one of the outcomes measured following either TORS therapy or CRT treatments. Amin et al (2023),^31^ examining tracheostomy requirements at 1 week, 6 weeks, and 6 months posttreatment, found no significant differences between the TORS group and the control cohort who underwent RT. This was most likely due to differences in local guidelines and was performed prophylactically to reduce the risk of hemorrhage and edema. A pooled analysis showed significantly lower gastrostomy tube usage at 6 and 12 months based on a common effects model, but not 3 months.

With regards to the length of usage, Hughes et al (2023) did not mention how long the tube was inserted, but instead stated no significant difference between gastrostomy tube prevalence and dependence between TORS alone, TORS with RT, and TORS with CRT treatment regimens. Those studies by Chen et al (2015), Sharma et al (2016), and Nichols et al (2019) did not specify the duration of tube usage in all cases.

### Pain Level

Three of the included studies reported on pain outcomes following TORS and control treatments, using a variety of pain scales.^13^, ^28^, ^36^ The specific metrics, time points, pain scores, and comparative statistics are described in Table 9. In a study conducted by Nichols et al,^13^ the TORS group was observed to have significantly lower pain levels than the RT group in the early postoperative period through both the EORTC QLQ‐C30 and EORTC QLQ‐H&N35 scale (*P* = .001). Ling et al^28^ was the only study reporting higher pain levels in the TORS group early postoperatively (at 1 month and 6 months) and lower levels later on (at 12 and 24 months) compared to CRT treatment, without disclosing significance levels. Scott et al^36^ found the TORS group to experience lower pain levels than their counterparts in both the initial and later stages postoperation compared to RT (with or without chemotherapy) albeit without significance levels, as measured by the EORTC H&N 35 scale.

**Table 9:** Pain‐Related Quality of Life Scores Following Transoral Robotic Surgery (TORS) Versus Control Groups

Author	Metric	Timescale	TORS ± adjuvant therapy	Control treatment	*P*‐value
Ling et al^28^	UW‐QOL version 4	1 mo	52 (27)	64 (24)	n.r.
		6 mo	81 (22)	83 (21)	n.r.
		12 mo	89 (23)	78 (27)	n.r.
		24 mo	86 (21)	82 (25)	n.r.
Nichols et al^13^	EORTC QLQ‐C30	1 y	21.8 (25.2)	8.0 (16.3)	.018
	EORTC H&N 35	1 y	13.3 (14.9)	9.0 (12.4)	.23
Scott et al^36^	EORTC H&N 35	Baseline	9.1 (11.7)	29.9 (19.9)	n.r.
		12 mo	8.9 (13.8)	11.1 (14.8)	n.r.
		Overall change	Mean change −0.3	Mean change −16.8	n.r.
			(*P* = .919)	(*P* = .037)	

Abbreviations: EORTC H&N 35, European Organisation for Research and Treatment of Cancer Head & Neck 35; EORTC QLQ‐C30, European Organisation for Research and Treatment of Cancer Core Quality of Life questionnaire; n.r., not reported; UW‐QOL, University of Washington Quality of Life questionnaire.

## Discussion

This systematic review aimed to assess the functional outcomes of TORS in comparison with the standard treatments of CRT for oropharyngeal cancers. The primary focus of the analysis was on OPSCC, which was the predominant cancer subtype among the studies reviewed. We included 16 studies in the analysis, and none of them contains a critical risk of bias.

### Baseline Characteristics and Cohort Matching

There were no significant differences in sex, age, tumor grade, or nodal grade between the TORS and CRT groups. Six studies matched the TORS and CRT cohorts by T stage,^13^, ^29^, ^30^, ^32^, ^35^, ^38^ while four studies matched them by N stage,^13^, ^30^, ^38^, ^39^ which adds some validity to their conclusion. Although different staging systems were used across studies, those focused on oropharyngeal cancer should show minimal variance between the American Joint Committee on Cancer (AJCC) 7th and 8th editions. However, p16 status remains a crucial factor due to its impact on nodal stage classification.

Three studies showed significant differences in tumor staging and five in nodal staging between TORS and CRT groups. Those discrepancies are expected, as the British Association of Head & Neck Oncologists (BAHNO) guidelines recommend transoral surgery for early‐stage T1/T2 N0‐1 cases only.^42^ Advanced tumors (T3/T4) and N2 diseases are generally managed by CRT due to the difficulty of achieving surgical margins. Moreover, N2 disease or extranodal extension often requires postoperative CRT, negating the benefits of TORS by resulting in triple modality treatment and increased morbidity.

### Variability in TORS Inclusion Criteria and the Role of Nodal Staging

The criteria for including and excluding patients from TORS varied considerably across studies, complicating comparisons and highlighting the lack of consensus on the appropriate nodal stage threshold for its use.

Ling et al (2016) offered TORS to patients with histology‐confirmed OPSCC and T0‐T2, N0‐N2 disease, considering adjuvant RT only for those with greater than or equal to N2 disease, angiolymphatic or perineural invasion, and <3 mm margins. Dhanireddy et al (2019) included patients up to N2b, recommending RT for N2a/N2b, perineural or lymphovascular invasion, and margins <2 mm.^28^ Nichols et al (2019) included patients with tumors up to T2 and N2 stages, restricting nodal inclusion to lymph nodes ≤4 cm without radiographic evidence of extranodal extension.^13^, ^36^ In contrast, Kaffenberger et al (2021) excluded patients early‐stage tumors (T1 and T2) treated with surgery alone and did not specify nodal stage selection.^37^

More recently, Hughes et al (2023) restricted inclusion to stage I‐II HPV‐associated OPSCC, excluding T3‐4 or N3 disease.^33^ Barbon et al (2022) excluded stage II (N2) disease when bilateral adenopathy (N2c) was present.^34^ Conversely, Meccariello et al (2020) included more advanced nodal disease (N2b, N2c, and N3).^38^ Genden et al (2011) reported that 38% of patients who would have otherwise required CRT for cT3‐4 or clinically node‐positive disease were able to avoid chemotherapy following TORS procedure, thereby reducing exposure to the adverse effects associated with chemotherapy agents.^39^ In contrast, Sharma et al (2016) conducted a retrospective cohort study without explicitly stating inclusion criteria based on tumor and nodal stages.^35^

In addition, More et al (2012) included patients with stage III or IVA oropharyngeal and supraglottic squamous cell carcinoma who had not received prior definitive treatment. Notably, 40% of patients (n = 8) with surgical margins <10 mm and no extracapsular spread avoided chemotherapy, thereby sparing them the associated toxicities.^32^ However, the inclusion of supraglottic tumors—generally considered suboptimal for TORS—illustrates further variability.

The broad variability underscores ongoing uncertainty about the optimal nodal stage cut‐off for TORS. While the United Kingdom Head and Neck Cancer National Multi‐disciplinary Guidelines advises RT with concurrent chemotherapy or consideration of tri‐modality treatment for most N2 cases,^42^ this is not a universally adopted standard. Instead, practices diverge internationally, influenced by resource availability, hospital protocols, and clinical judgment. Ultimately, the decision to proceed with TORS should consider not only nodal staging but also tumor characteristics, patient comorbidities, and preferences. The variability in study inclusion criteria reflects this complexity and highlights the need for more standardized, evidence‐based guidelines.

### HPV/p16 Status and Its Reporting

There was a mixed patient demographic regarding p16/HPV status. Although 11 studies reported patient demographics, none presented stratified results, hindering further meta‐analysis. Additionally, no study performed a stratified analysis based on p16/HPV status in relation to functional outcomes.

HPV p16 immunochemistry is a simple yet highly informative prognostic biomarker. The 2024 BAHNO Guidelines highlighted its importance in staging and counseling, though not in amending the treatment protocol.^42^ Despite that, the AJCC 8th edition integrates p16 status into staging, adding an extra layer of patient stratification and evaluation of treatment outcomes.

The HPV‐driven OPSCC typically occurs in younger and healthier individuals with fewer comorbidities and lower exposure to high‐risk social factors. These tumors exhibit high radiosensitivity and are associated with significantly improved clinical outcomes. As a result, treatment protocols are increasingly being de‐escalated, creating opportunities to consider single‐modality surgical approaches, including minimally invasive techniques such as TORS.

### Swallowing Function

Swallowing outcomes varied across studies. Some studies reported improvements in appetite^30^, ^36^ and increased saliva production^28^ following TORS, while others observed no significant differences between both groups across these metrics.^27^, ^32^, ^34^, ^37^, ^40^ The authors faced significant challenges in conducting a comprehensive comparative analysis of swallowing outcomes due to the wide variability in assessment tools employed across studies. Instruments used included the MDADI, UW‐QOL, EORTC QLQ‐C30, and EAT‐10.

Furthermore, the timing of outcome evaluations varied considerably, with follow‐up durations ranging from as early as 1 week to as long as 2 years, further complicating cross‐study comparisons. The meta‐analysis revealed no significant differences in MDADI swallowing scores between TORS and CRT at baseline or at 3 to 6 months posttreatment, suggesting that patients may have similar baseline function and early recovery trajectories in both treatment arms. The substantial heterogeneity observed across studies may be attributable to patient demographics and treatment protocols. Notably, the pooled estimate at 12 months demonstrated a statistically significant difference in favor of CRT at 12 months, indicating that CRT may offer a more favorable trajectory for swallowing function.

However, outcomes measured at 3, 6, and 12 months may not adequately reflect the long‐term impact of pharyngeal RT, which can progressively impair swallowing function over several years. The delayed side effects, including fibrosis, xerstomia, and pharyngeal strictures,^43^ indicate the importance of extended follow‐up on evaluating swallowing outcomes.

### Gastrostomy Tube Use

Gastrostomy tube use also varied significantly in timing and reporting. The meta‐analysis revealed substantial heterogeneity at 3 and 6 months, which markedly decreased by 12 months, at which point TORS was associated with a statistically significant reduction in gastrostomy tube dependence.

Given that the TORS procedure typically lasts a few hours, while RT is administered over 6 to 7 weeks, the more relevant clinical endpoint is ongoing tube use at defined follow‐up intervals (eg, 3 or 6 months), rather than initial insertion. The delayed effect of RT on swallowing function^44^ further complicates the interpretation of tube dependence over time. The heterogeneity of institution protocol on gastrostomy tube placement and clinical judgment poses challenges for impactful cross‐study comparisons. The relative novelty of TORS contributes to the limited number of high‐quality comparative studies. As robotic technologies become more widely adopted and clinical experience grows, future analyses may yield more robust and generalizable conclusions.

### Limitations and Future Directions

The scope of this meta‐analysis was limited in terms of the number of included studies, with only three reporting swallowing function (MDADI) and two reporting pain at the 12‐month endpoint. Also, evidence comparing the functional outcomes of TORS, transoral laser microsurgery, and other endoscopic resections is currently limited. Larger studies with more comprehensive patient data are needed to better define these differences.

The TORS group typically has lower‐stage disease due to selection bias, as patients with T1, T2, and select T3 tumors are primarily chosen for surgery. In contrast, advanced OPSCC (T3‐4 or N2+) is typically managed with RT or CRT, unless tri‐modality treatment is selected following patient counseling. Notably, only the study by Amin et al (2023) used stage‐matched CRT groups.^31^

As TORS continues to evolve, ongoing research is essential to better define its benefits over conventional treatments. Evaluating the impact of different techniques and instruments within TORS will be key to establishing standardized protocols that ensure optimal patient outcomes. Moreover, comparative studies evaluating the efficacy of TORS versus RT or CRT across different tumor stages are needed, especially given the current emphasis on early‐stage disease (T0‐T2, N0‐N2), which likely reflects TORS's limited suitability for more advanced cases.^45^

## Conclusion

This review provides the most comprehensive analysis to date of functional outcomes in trials comparing TORS and CRT. TORS shows promise for selected tumors, but methodological inconsistencies, particularly in eligibility criteria and HPV p16 status, limit comparability. Standardized guidelines and further trials are needed to inform treatment choice.

## Author Contributions

**Simpson Shiu Chung Tam**, design conception, methodology development, data curation and analysis, investigation, and manuscript development; **Nihal Sogandji**, methodology development, data curation and analysis, investigation, and manuscript development; **Muzammil Arif Din Abdul Jabbar**, design conception, methodology development, data curation and analysis, investigation, manuscript development, and research supervision; **Shazia Huma Absar**, data curation and analysis, investigation, and manuscript development; **Mikesh Kalpesh Patel**, data curation and analysis, investigation, and manuscript development; **Jeffrey Tooze**, data curation and analysis, investigation, and manuscript development; **Eleanor Barker**, methodology development, data curation and analysis, and manuscript development; **Manaf Khatib**, methodology development, manuscript development, and research supervision; **Anant Patel**, manuscript development and research supervision; **George Mochloulis**, manuscript development and research supervision; **Amit Gupta**, manuscript development and research supervision.

## Disclosures

### Competing interests

The authors have no competing interests to declare that are relevant to the content of this article.

### Funding source

The open‐access publication is made available by the open‐access agreements under the Joint Information Systems Committee – University of Cambridge. The funder has no role in study design; data collection, analysis, and interpretation; the writing of the manuscript; and the decision to submit the manuscript for publication.

## Supporting information

Supporting Information.

Supporting Information.
