# Supplementary material for: A Meta‐analysis of Functional Outcomes and Recovery Metrics Comparing Transoral Robotic Surgery and (Chemo)Radiotherapy
Source: Otolaryngol Head Neck Surg. 2025 Dec 7;174(1):57–76. doi: 10.1002/ohn.70069 (PMC12794744; doi:10.1002/ohn.70069)
Supplement: Supplementary file 1 — Supporting Information. [file OHN-174-57-s002.docx]

Supplementary Document

Table of Contents

[Section 1 – Search Strategy 2](#_Toc198597804)

[Section 2 – Risk of Bias Assessment 8](#_Toc198597805)

[Section 3 – Extended GRADE Evidence Profiles 28](#_Toc198597806)

[Section 4 – Comparative Analysis of Swallowing Function Outcomes 59](#_Toc198597807)

# **Section 1 – Search Strategy**

The databases Medline (via Ovid), Embase (via Ovid), Cochrane Library and Web of Science (Core Collection) were searched from 1997 to September 2023 by EB. The search strategy was peer-reviewed by two librarian colleagues of EB using the Peer Review of Electronic Search Strategies (PRESS) checklist (McGowan et al., 2016), and evaluated against the PRISMA-S guidelines (Rethlefsen et al., 2021). The completed PRISMA-S checklist is shown in the supplementary material. Databases was searched by EB separately, rather than multiple databases being searched on the same platform. The search syntax was adapted for each database, and to account for variation between thesaurus terms/controlled vocabulary across each database. Results were imported to Endnote 21 by EB for deduplication, using the method outlined by Bramer et al. (Bramer et al., 2016). Grey literature searches were carried out in WHO Clinical Trials Registry, ScanMedicine, NIHR Be Part of Research, Clinicaltrails.gov, ETHOS and a Google search. Manual searching involved looking back through the tables of contents of the Journal of Craniofacial Surgery (2000-present) and Journal of Robotic Surgery (2007-present). The review team scanned reference lists of included articles and track the citations of included papers. Dates when searches were run were indicated in the results table. The full search strategies used in each database are documented in full below.

**Table S1.**
**Database Search Results and Deduplication Summary (All searched on 25th September 2023)**

**Legend:**
The table summarises the number of records retrieved from each database before and after deduplication. Searches were conducted in Medline, Embase, Cochrane Library, and Web of Science on 25th September 2023. A total of 14,365 records were initially retrieved. After removing duplicates, 9,612 unique records remained for screening.

| Database (All searched 25^th^ September 2023) | Total before deduplication | Total after deduplication |
| --- | --- | --- |
| Medline | 3962 |  |
| Embase | 4267 |  |
| Cochrane | 2245 |  |
| Web of Science | 3891 |  |
| **Total** | 14365 | 9612 |

**Ovid MEDLINE(R) and Epub Ahead of Print, In-Process, In-Data-Review & Other Non-Indexed Citations, Daily and Versions <1946 to September 22, 2023> Searched 25^th^ September 2023**

1 (cancer* or carcinoma* or neoplasm* or malignan* or tumor* or tumour* or oncolog* or sarcoma* or metasta* or lesion*).ti,ab,kw,kf. 4790035

2 ((abnormal or pathological) adj (growth* or mass*)).ti,ab,kw,kf. 2665

3 1 or 2 4791713

4 (craniofacial or facial or head or neck or ear or larynx or laryngeal or pharynx or pharyngeal or oropharyngeal or parapharyngeal or nasal or sinonasal or nose or nasopharyngeal or otorhinolaryng* or otolaryng* or throat or skull or face or jaw or maxillofacial or cervicofacial or upper cervical or cranial or oral or palate or tonsil or lip or salivary gland* or tongue or esophag* or oesophag*).ti,ab,kw,kf. 2345794

5 3 and 4 554530

6 exp "Head and Neck Neoplasms"/ 352708

7 5 or 6 695671

8 ((robot* or mechani* or automat* or technology assist* or computer enhanc* or computer assist* or computer guided or computer aid* or imag* guided or single port) adj3 (surg* or operat* or procedure*)).ti,ab,kw,kf. 56068

9 tors.ti,ab,kw,kf. 1100

10 surgery, computer-assisted/ or robotic surgical procedures/ 36479

11 8 or 9 or 10 79488

12 7 and 11 4252

13 limit 12 to (yr="1997 -Current" and (chinese or english or spanish)) 3962

**Embase <1974 to 2023 September 22> Searched 25^th^ September 2023**

1 (cancer* or carcinoma* or neoplasm* or malignan* or tumor* or tumour* or oncolog* or sarcoma* or metasta* or lesion*).ti,ab,kw. 6308126

2 ((abnormal or pathological) adj (growth* or mass*)).ti,ab,kw. 3455

3 1 or 2 6310195

4 (craniofacial or facial or head or neck or ear or larynx or laryngeal or pharynx or pharyngeal or oropharyngeal or parapharyngeal or nasal or sinonasal or nose or nasopharyngeal or otorhinolaryng* or otolaryng* or throat or skull or face or jaw or maxillofacial or cervicofacial or upper cervical or cranial or oral or palate or tonsil or lip or salivary gland* or tongue or esophag* or oesophag*).ti,ab,kw. 2991066

5 3 and 4 778901

6 exp *"head and neck tumor"/ 269159

7 5 or 6 876658

8 ((robot* or mechani* or automat* or technology assist* or computer enhanc* or computer assist* or computer guided or computer aid* or imag* guided or single port) adj3 (surg* or operat* or procedure*)).ti,ab,kw. 73186

9 tors.ti,ab,kw. 2520

10 exp *computer assisted surgery/ 23248

11 8 or 9 or 10 87215

12 7 and 11 4525

13 limit 12 to ((chinese or english or spanish) and yr="1997 -Current") 4267

**Cochrane searched 25^th^ September 2023**

#1 (cancer* or carcinoma* or neoplasm* or malignan* or tumor* or tumour* or oncolog* or sarcoma* or metasta* or lesion*):ti,ab,kw (Word variations have been searched) 308041

#2 ((abnormal or pathological) NEXT (growth* or mass*)):ti,ab,kw (Word variations have been searched) 81

#3 #1 or #2 308095

#4 (craniofacial or facial or head or neck or ear or larynx or laryngeal or pharynx or pharyngeal or oropharyngeal or parapharyngeal or nasal or sinonasal or nose or nasopharyngeal or otorhinolaryng* or otolaryng* or throat or skull or face or jaw or maxillofacial or cervicofacial or upper cervical or cranial or oral or palate or tonsil or lip or salivary gland* or tongue or esophag* or oesophag*):ti,ab,kw (Word variations have been searched) 372760

#5 #3 and #4 67837

#6 MeSH descriptor: [Head and Neck Neoplasms] explode all trees 8216

#7 #5 or #6 68537

#8 ((robot* or mechani* or automat* or technology assist* or computer enhanc* or computer assist* or computer guided or computer aid* or imag* guided or single port) NEAR/3 (surg* or operat* or procedure*)):ti,ab,kw (Word variations have been searched) 54703

#9 (tors):ti,ab,kw (Word variations have been searched) 606

#10 MeSH descriptor: [Surgery, Computer-Assisted] this term only 960

#11 MeSH descriptor: [Robotic Surgical Procedures] this term only 694

#12 #8 or #9 or #10 or #11 55249

#13 #7 and #12 2245 (25 Cochrane reviews (no limits applied), 2220 Trials (Year custom range limited to 1997-2023))

**# Database: Web of Science Core Collection Searched 25^th^ September 2023**

# Entitlements:

- WOS.IC: 1993 to 2023

- WOS.CCR: 1985 to 2023

- WOS.SCI: 1900 to 2023

- WOS.AHCI: 1975 to 2023

- WOS.BHCI: 2008 to 2023

- WOS.BSCI: 2008 to 2023

- WOS.ESCI: 2018 to 2023

- WOS.ISTP: 1990 to 2023

- WOS.SSCI: 1956 to 2023

- WOS.ISSHP: 1990 to 2023

# Searches:

1: cancer* or carcinoma* or neoplasm* or malignan* or tumor* or tumour* or oncolog* or sarcoma* or metasta* or lesion* (Topic) Date Run: Mon Sep 25 2023 11:30:04 GMT+0100 (British Summer Time) Results: 5667177

2: (abnormal or pathological) NEAR/0 (growth* or mass*) (Topic) Date Run: Mon Sep 25 2023 11:30:25 GMT+0100 (British Summer Time) Results: 3385

3: #1 OR #2 Date Run: Mon Sep 25 2023 11:30:39 GMT+0100 (British Summer Time) Results: 5669462

4: craniofacial or facial or head or neck or ear or larynx or laryngeal or pharynx or pharyngeal or oropharyngeal or parapharyngeal or nasal or sinonasal or nose or nasopharyngeal or otorhinolaryng* or otolaryng* or throat or skull or face or jaw or maxillofacial or cervicofacial or "upper cervical" or cranial or oral or palate or tonsil or lip or "salivary gland*" or tongue or esophag* or oesophag* (Topic) Date Run: Mon Sep 25 2023 11:31:18 GMT+0100 (British Summer Time) Results: 3510279

5: #3 AND #4 Date Run: Mon Sep 25 2023 11:31:51 GMT+0100 (British Summer Time) Results: 604807

6: (robot* or mechani* or automat* or "technology assist*" or "technology-assist*" or "computer enhanc*" or "computer-enhanc*" or "computer assist*" or "computer-assist*" or "computer guided" or "computer-guided" or "computer aid*" or "computer-aid*" or "imag* guided" or "image-guided" or “single port” or “single-port”) NEAR/3 (surg* or operat* or procedure*) (Topic) Date Run: Mon Sep 25 2023 11:32:51 GMT+0100 (British Summer Time) Results: 137851

7: tors (Topic) Date Run: Mon Sep 25 2023 11:33:09 GMT+0100 (British Summer Time) Results: 20363

8: #6 OR #7 Date Run: Mon Sep 25 2023 11:33:33 GMT+0100 (British Summer Time) Results: 157260

9: #5 AND #8 Date Run: Mon Sep 25 2023 11:33:53 GMT+0100 (British Summer Time) Results: 4098

10: #5 AND #8 and 2023 or 2022 or 2021 or 2020 or 2019 or 2018 or 2017 or 2016 or 2015 or 2014 or 2013 or 2012 or 2011 or 2010 or 2009 or 2008 or 2007 or 2006 or 2005 or 2004 or 2003 or 2002 or 2001 or 2000 or 1999 or 1998 or 1997 (Publication Years) Date Run: Mon Sep 25 2023 11:34:39 GMT+0100 (British Summer Time) Results: 4033

11: #5 AND #8 and 2023 or 2022 or 2021 or 2020 or 2019 or 2018 or 2017 or 2016 or 2015 or 2014 or 2013 or 2012 or 2011 or 2010 or 2009 or 2008 or 2007 or 2006 or 2005 or 2004 or 2003 or 2002 or 2001 or 2000 or 1999 or 1998 or 1997 (Publication Years) and English or Spanish or Chinese (Languages) Date Run: Mon Sep 25 2023 11:35:03 GMT+0100 (British Summer Time) Results: 3891

**Grey literature searches**

WHO Clinical trials registry platform searched “transoral robotic surgery”

<https://trialsearch.who.int/Default.aspx> searched 3rd October 2023, 42 results.

Google advanced search, “transoral robotic surgery”

ScanMedicine searched “transoral robotic surgery”.

<https://scanmedicine.com/> searched 3^rd^ October 2023, 55 results.

NIHR Be part of research searched “transoral robotic surgery”

<https://bepartofresearch.nihr.ac.uk/> searched 3^rd^ October 2023, 2 results

Clinical trials.gov searched transoral robotic surgery as an intervention

<https://clinicaltrials.gov/> searched 3^rd^ October 2023, 52 results

Ethos searched transoral robotic surgery

<https://ethos.bl.uk/> searched 3^rd^ October 2023, 2 results

**References:**

BRAMER, W. M., GIUSTINI, D., DE JONGE, G. B., HOLLAND, L. & BEKHUIS, T. 2016. De-duplication of database search results for systematic reviews in EndNote. J Med Libr Assoc, 104**,** 240-3.

MCGOWAN, J., SAMPSON, M., SALZWEDEL, D. M., COGO, E., FOERSTER, V. & LEFEBVRE, C. 2016. PRESS peer review of electronic search strategies: 2015 guideline statement. Journal of clinical epidemiology, 75**,** 40-46.

RETHLEFSEN, M. L., KIRTLEY, S., WAFFENSCHMIDT, S., AYALA, A. P., MOHER, D., PAGE, M. J. & KOFFEL, J. B. 2021. PRISMA-S: an extension to the PRISMA statement for reporting literature searches in systematic reviews. Systematic reviews, 10**,** 1-19.

**#Google Search on 12^th^ October 2023**

MD searched on Google with the term 'transoral robotic surgery' and received 146 results.

# **Section 2 – Risk of Bias Assessment**

A structured risk of bias assessment was conducted for each included non-randomised study using the ROBINS-I tool, which evaluates bias across seven domains: confounding, selection of participants, classification of interventions, deviations from intended interventions, missing data, measurement of outcomes, and selection of the reported result. Each study was rated for risk of bias in each domain and assigned an overall risk of bias rating. The majority of studies were judged to have a moderate risk of bias, primarily due to concerns related to confounding and outcome measurement. One study included in the analysis was a randomised controlled trial (RCT) and was assessed separately using the Cochrane ROB-2 tool, which evaluates bias specific to the design and conduct of randomised trials. These assessments inform the GRADE evaluations and provide essential context for interpreting the summary effect estimates.

**Table S2.**
**Risk of Bias Assessment Across Included Non-Randomised Studies (ROBINS-I Tool)**

**Legend:**
The table presents the overall risk of bias ratings for the included studies. Each study was evaluated using a standardised tool, and the level of bias was categorised as *Low*, *Moderate*, or *Serious*. These assessments help determine the quality and reliability of the evidence contributed by each study.

| **Study (Year)** | **Overall Bias** |
| --- | --- |
| Amin et al. (2023) | Moderate |
| Barbon et al. (2021) | Moderate |
| Hutcheson et al. (2019) | Moderate |
| Barbon et al. (2022) | Low |
| Chen et al. (2015) | Moderate |
| Dhanireddy et al. (2019) | Low |
| Genden et al. (2011) | Serious |
| Hughes et al. (2023) | Moderate |
| Kaffenberger et al. (2021) | Serious |
| Ling et al. (2016) | Low |
| Meccariello et al. (2020) | Serious |
| More et al. (2013) | Moderate |
| Scott et al. (2021) | Serious |
| Scott et al. (2023) | Serious |
| Sharma et al. (2016) | Moderate |


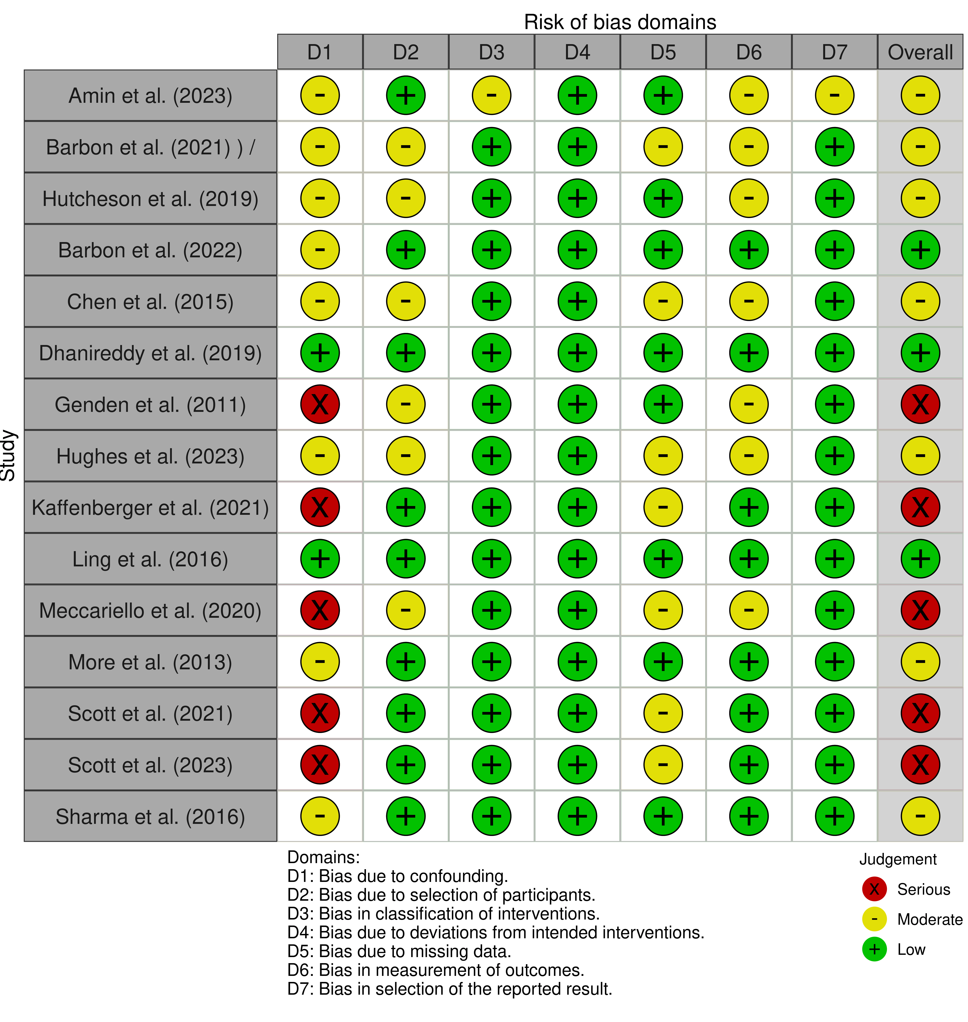


**Figure S1. Risk of bias assessment (ROBINS-I) across seven domains for included studies.** Colour-coding indicates low, moderate, or serious risk; rightmost column shows overall bias judgment.


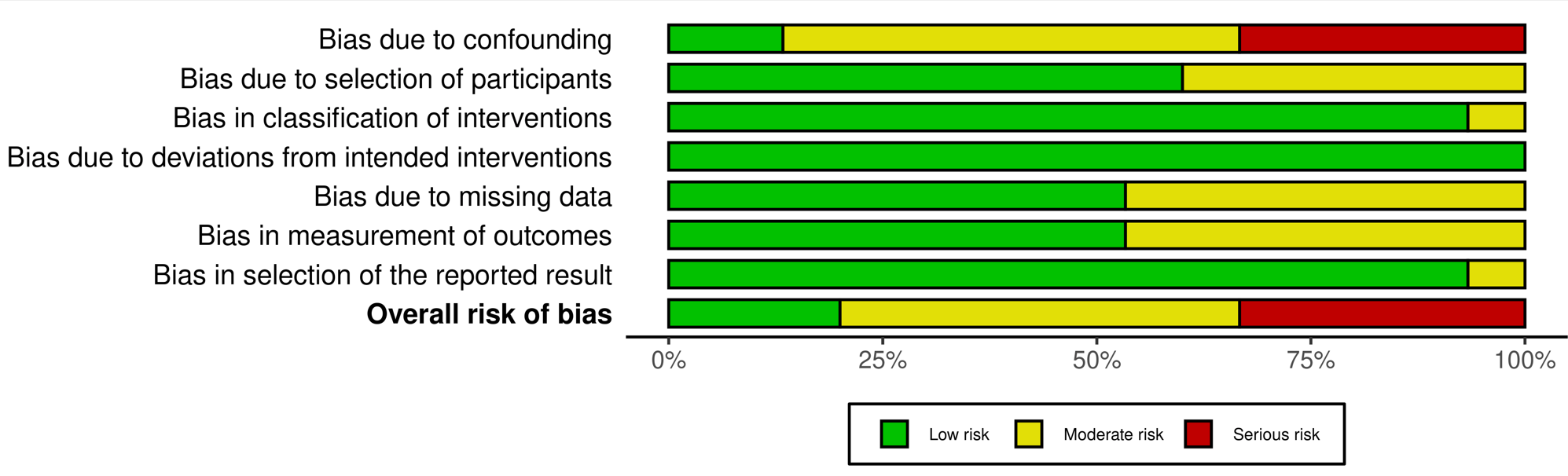


**Figure S2. Proportional distribution of risk of bias (ROBINS-I) across seven domains.** Bars show percentages of studies with low, moderate, or serious risk; bottom bar indicates overall bias.

**Table S3.**
**Overall Risk of Bias Judgements for Randomised Controlled Trials (ROB-2 Tool)**

**Legend:**
The table summarises the overall risk of bias for the included randomised controlled trial, assessed using the Cochrane Risk of Bias 2 (ROB-2) tool. The judgement *“Some Concerns”* indicates that the study had issues in one or more domains that may affect the reliability of the result, but not to a degree warranting a high risk of bias classification.

| **Study (Year)** | **Overall Bias** |
| --- | --- |
| Nichols et al 2019 | Some Concerns |


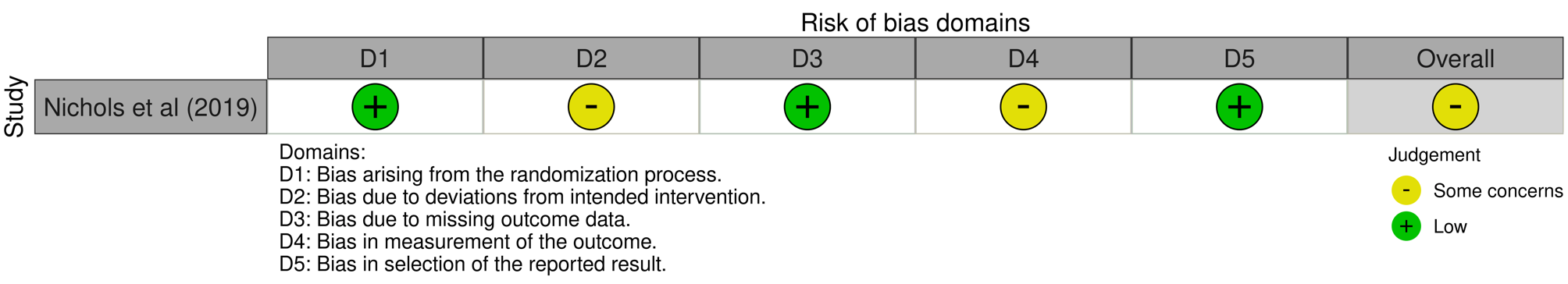


**Figure S3. Risk of bias assessment for Nichols et al. (2019) using ROB-2 tool.** Five domains rated low risk or some concerns; overall judgement reflects combined domain ratings.


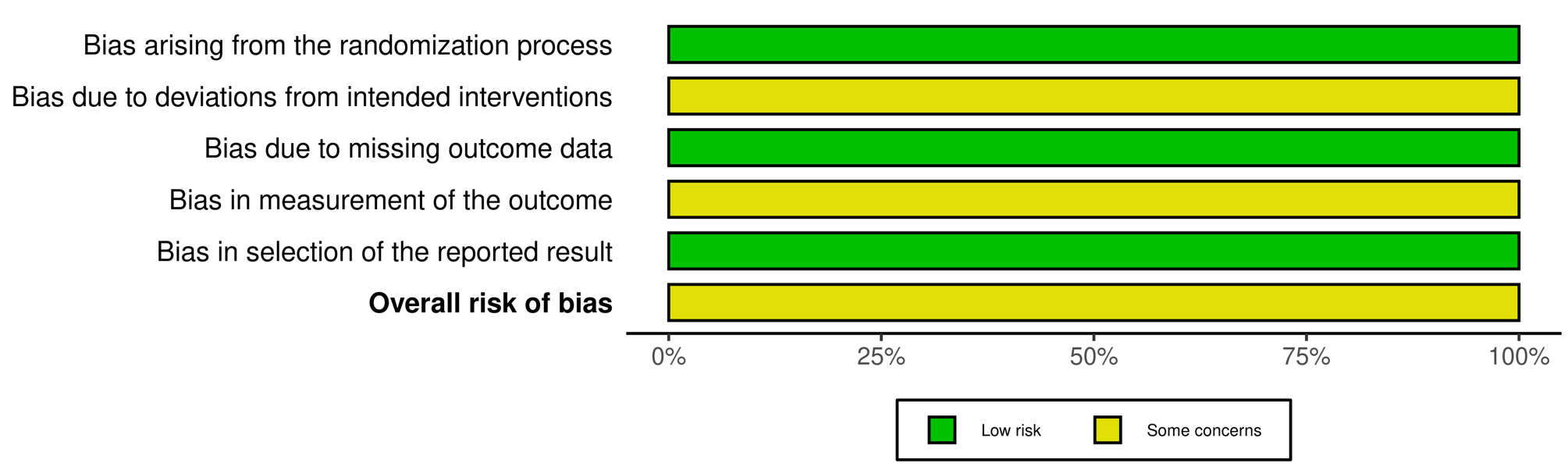


**Figure S4. Proportional distribution of risk of bias judgements for Nichols et al. (2019) using ROB-2 tool.** Bars show domain-level ratings; overall judgement indicates some concerns.

**A. Non-Randomised Studies (ROBINS-I Tool)**

**Table S4.**

**Assessment of Bias Due to Confounding Across Included Studies (ROBINS-I Tool)**

**Legend:**

This table details the assessment of **bias due to confounding** across all included studies using a structured checklist of eight signalling questions. Each response is coded as follows:

- **Y** = Yes
- **N** = No
- **PY** = Probably Yes
- **PN** = Probably No
- **NA** = Not Applicable

**Overall Bias Judgement** is categorised as **Low**, **Moderate**, or **Serious** based on the aggregate of responses.

|  | **Bias due to confounding** | | | | | | | | |
| --- | --- | --- | --- | --- | --- | --- | --- | --- | --- |
| **Study (Year)** | 1.1 Is there potential for confounding of the effect of intervention in this study? | 1.2 Was the analysis based on splitting participants' follow up time according to intervention received? | 1.3 Were intervention discontinuations or switches likely to be related to factors that are prognostic for the outcome? | 1.4 Did the authors use an appropriate analysis method that controlled for all the important confounding domains? | 1.5 Were confounding domains that were controlled for measured validly and reliably by the variables available in this study? | 1.6 Did the authors control for any post-intervention variables that could have been affected by the intervention? | 1.7 Did the authors use an appropriate analysis method that adjusted for all the important confounding domains and for time-varying confounding? | 1.8 Were confounding domains that were adjusted for measured validly and reliably by the variables available in this study? | Overall |
| Amin et al. (2023) | Y | N | NA | Y | Y | N | PY | Y | Moderate |
| Barbon et al. (2021) | Y | N | NA | Y | Y | PN | PN | NA | Moderate |
| Hutcheson et al. (2019) | Y | N | NA | Y | Y | NA | N | NA | Moderate |
| Barbon et al. (2022) | Y | N | NA | Y | Y | N | Y | Y | Moderate |
| Chen et al. (2015) | Y | N | N | PY | Y | N | Y | Y | Moderate |
| Dhanireddy et al. (2019) | Y | N | N | Y | Y | N | Y | Y | Low |
| Genden et al. (2011) | Y | N | NA | PY | NA | PN | N | NA | Serious |
| Hughes et al. (2023) | Y | N | NA | Y | Y | PN | PN | NA | Moderate |
| Kaffenberger et al. (2021) | Y | N | N | N | NA | N | N | NA | Serious |
| Ling et al. (2016) | Y | N | N | Y | Y | N | Y | Y | Low |
| Meccariello et al. (2020) | Y | N | NA | N | NA | PN | N | NA | Serious |
| More et al. (2013) | Y | N | N | PY | Y | N | Y | Y | Moderate |
| Scott et al. (2021) | Y | N | N | N | N | N | N | NA | Serious |
| Scott et al. (2023) | Y | N | N | N | NA | N | N | NA | Serious |
| Sharma et al. (2016) | Y | N | N | PY | Y | N | Y | Y | Moderate |

**Table S5.**

**Assessment of Bias in Selection of Participants into the Study (ROBINS-I Tool)**

**Legend:**

This table presents the risk of bias judgements **concerning selection of participants into the** study or analysis, based on five signalling questions. Each item is assessed according to structured guidance, with responses coded as:

- **Y** = Yes
- **N** = No
- **PY** = Probably Yes
- **PN** = Probably No
- **NI** = No Information
- **NA** = Not Applicable

The **Overall Bias** rating (Low, Moderate) reflects the cumulative impact of these factors on study validity.

|  | **Bias in selection of participants into the study** | | | | | |
| --- | --- | --- | --- | --- | --- | --- |
| **Study (Year)** | 2.1 Was selection of participants into the study (or into the analysis) based on participant characteristics observed after the start of intervention? | 2.2 Were the post-intervention variables that influenced selection likely to be associated with intervention? | 2.3 Were the post-intervention variables that influenced selection likely to be influenced by the outcome or a cause of the outcome? | 2.4 Do start of follow-up and start of intervention coincide for most participants? | 2.5 Were adjustment techniques used that are likely to correct for the presence of selection biases? | Overall |
| Amin et al. (2023) | N | NA | NA | Y | NA | Low |
| Barbon et al. (2021) | N | NA | NA | PN | NA | Moderate |
| Hutcheson et al. (2019) | N | NA | NA | NI | NA | Moderate |
| Barbon et al. (2022) | N | NA | NA | Y | NA | Low |
| Chen et al. (2015) | N | NA | NA | PY | NA | Moderate |
| Dhanireddy et al. (2019) | N | NA | NA | Y | NA | Low |
| Genden et al. (2011) | N | NA | NA | PY | NA | Moderate |
| Hughes et al. (2023) | N | NA | NA | PN | NA | Moderate |
| Kaffenberger et al. (2021) | N | NA | NA | Y | NA | Low |
| Ling et al. (2016) | N | NA | NA | y | NA | Low |
| Meccariello et al. (2020) | N | NA | NA | NI | N | Moderate |
| More et al. (2013) | N | NA | NA | Y | NA | Low |
| Scott et al. (2021) | N | NA | NA | Y | NA | Low |
| Scott et al. (2023) | N | NA | NA | Y | NA | Low |
| Sharma et al. (2016) | N | NA | NA | Y | NA | Low |

**Table S6.**

**Assessment of Bias in Classification of Interventions (ROBINS-I Tool)**

**Legend:**

This table presents the risk of bias judgements concerning classification of interventions across the study, based on three signalling questions. Each item is assessed according to structured guidance, with responses coded as:

- **Y** = Yes
- **N** = No
- **PY** = Probably Yes
- **PN** = Probably No
- **NI** = No Information
- **NA** = Not Applicable

The **Overall** judgement reflects the cumulative evaluation across these questions and is categorised as low or moderate risk of bias.

|  | **Bias in classification of interventions** | | | |
| --- | --- | --- | --- | --- |
| **Study (Year)** | 3.1 Were intervention groups clearly defined? | 3.2 Was the information used to define intervention groups recorded at the start of the intervention? | 3.3 Could classification of intervention status have been affected by knowledge of the outcome or risk of the outcome? | Overall |
| Amin et al. (2023) | Y | N | PN | Moderate |
| Barbon et al. (2021) | Y | Y | N | Low |
| Hutcheson et al. (2019) | Y | Y | N | Low |
| Barbon et al. (2022) | Y | Y | N | Low |
| Chen et al. (2015) | Y | Y | N | Low |
| Dhanireddy et al. (2019) | Y | Y | N | Low |
| Genden et al. (2011) | Y | Y | N | Low |
| Hughes et al. (2023) | Y | Y | N | Low |
| Kaffenberger et al. (2021) | Y | Y | N | Low |
| Ling et al. (2016) | Y | Y | N | Low |
| Meccariello et al. (2020) | Y | Y | N | Low |
| More et al. (2013) | Y | Y | N | Low |
| Scott et al. (2021) | Y | Y | N | Low |
| Scott et al. (2023) | Y | Y | N | Low |
| Sharma et al. (2016) | Y | Y | N | Low |

**Table S7.**

**Assessment of Risk of Bias Due to Deviations from Intended Interventions (ROBINS-I Tool)**

**Legend:**

This table presents domain-specific judgements for **bias due to deviations from intended interventions**, assessed using the ROBINS-I tool. Each column contains the full wording of the signalling questions used to evaluate potential bias. All studies were judged to be at **low risk** in this domain.

**Response codes:**

- **Y** = Yes
- **N** = No
- **NA** = Not Applicable

The assessments reflect whether studies maintained intervention fidelity and used appropriate methods to analyse the effect of adherence.

|  | **Bias due to deviations from intended interventions** | | | | | | |
| --- | --- | --- | --- | --- | --- | --- | --- |
| **Study (Year)** | 4.1 Were there deviations from the intended intervention beyond what would be expected in usual practice? | 4.2 Were these deviations from intended intervention unbalanced between groups *and* likely to have affected the outcome? | 4.3 Were important co-interventions balanced across intervention groups? | 4.4 Was the intervention implemented successfully for most participants? | 4.5 Did study participants adhere to the assigned intervention regime? | 4.6 Was an appropriate analysis used to estimate the effect of starting and adhering to the intervention? | Overall |
| Amin et al. (2023) | N | NA | NA | NA | NA | NA | Low |
| Barbon et al. (2021) | N | NA | Y | Y | Y | NA | Low |
| Hutcheson et al. (2019) | N | NA | Y | Y | Y | NA | Low |
| Barbon et al. (2022) | N | NA | NA | NA | NA | NA | Low |
| Chen et al. (2015) | N | NA | Y | Y | Y | NA | Low |
| Dhanireddy et al. (2019) | N | NA | NA | NA | NA | NA | Low |
| Genden et al. (2011) | N | NA | Y | Y | Y | NA | Low |
| Hughes et al. (2023) | N | NA | Y | Y | Y | NA | Low |
| Kaffenberger et al. (2021) | N | NA | NA | NA | NA | NA | Low |
| Ling et al. (2016) | N | NA | NA | NA | NA | NA | Low |
| Meccariello et al. (2020) | N | NA | Y | Y | Y | NA | Low |
| More et al. (2013) | N | NA | NA | NA | NA | NA | Low |
| Scott et al. (2021) | N | NA | NA | NA | NA | NA | Low |
| Scott et al. (2023) | N | NA | NA | NA | NA | NA | Low |
| Sharma et al. (2016) | N | NA | NA | NA | NA | NA | Low |

**Table S8.**

**Assessment of Risk of Bias Due to Missing Data (ROBINS-I Tool)**

**Legend:**

This table presents the domain-specific risk of bias assessments related to **missing data**, using the **ROBINS-I** framework. Each signalling question was answered based on the information provided in each study.

**Response codes:**

- **Y** = Yes
- **N** = No
- **PY** = Probably Yes
- **PN** = Probably No
- **NA** = Not Applicable

The **Overall** risk of bias rating reflects the cumulative impact of responses, classified as **Low** or **Moderate**.

|  | **Bias due to missing data** | | | | | |
| --- | --- | --- | --- | --- | --- | --- |
| **Study (Year)** | 5.1 Were outcome data available for all, or nearly all, participants? | 5.2 Were participants excluded due to missing data on intervention status? | 5.3 Were participants excluded due to missing data on other variables needed for the analysis? | 5.4 Are the proportion of participants and reasons for missing data similar across investigations? | 5.5 Is there evidence that results were robust to the presence of missing data? | Overall |
| Amin et al. (2023) | Y | N | N | NA | NA | Low |
| Barbon et al. (2021) | Y | N | PY | Y | PN | Moderate |
| Hutcheson et al. (2019) | Y | N | N | NA | NA | Low |
| Barbon et al. (2022) | Y | N | N | NA | NA | Low |
| Chen et al. (2015) | Y | N | PY | Y | PN | Moderate |
| Dhanireddy et al. (2019) | Y | N | N | NA | NA | Low |
| Genden et al. (2011) | Y | N | N | NA | NA | Low |
| Hughes et al. (2023) | Y | N | PY | Y | PN | Moderate |
| Kaffenberger et al. (2021) | PY | N | Y | PN | NA | Moderate |
| Ling et al. (2016) | Y | N | N | NA | NA | Low |
| Meccariello et al. (2020) | PY | Y | N | NA | NA | Moderate |
| More et al. (2013) | Y | N | N | NA | NA | Low |
| Scott et al. (2021) | Y | N | Y | N | N | Moderate |
| Scott et al. (2023) | PN | N | PY | Y | PN | Moderate |
| Sharma et al. (2016) | Y | N | N | NA | NA | Low |

**Table S9.**

**Assessment of Risk of Bias in Measurement of Outcomes (ROBINS-I Tool)**

**Legend:**

This table presents the assessment of **bias in measurement of outcomes**, using signalling questions from the **ROBINS-I** tool. This domain examines the objectivity and reliability of outcome measurement across included studies.

**Response codes:**

- **Y** = Yes
- **N** = No
- **PY** = Probably Yes
- **PN** = Probably No

The **Overall** judgement reflects the study's susceptibility to bias in outcome measurement and is rated as **Low**, **Moderate**, or **Serious**.

|  | **Bias in measurement of outcomes** | | | | |
| --- | --- | --- | --- | --- | --- |
| **Study (Year)** | 6.1 Could the outcome measure have been influenced by knowledge of the intervention received? | 6.2 Were outcome assessors aware of the intervention received by study participants? | 6.3 Were the methods of outcome assessment comparable across intervention groups? | 6.4 Were any systematic errors in measurement of the outcome related to intervention received? | Overall |
| Amin et al. (2023) | PN | Y | Y | N | Moderate |
| Barbon et al. (2021) | PY | Y | Y | N | Moderate |
| Hutcheson et al. (2019) | PY | PY | PY | PN | Moderate |
| Barbon et al. (2022) | N | Y | Y | N | Low |
| Chen et al. (2015) | PY | Y | Y | N | Moderate |
| Dhanireddy et al. (2019) | N | Y | Y | N | Low |
| Genden et al. (2011) | PY | PY | PY | N | Moderate |
| Hughes et al. (2023) | PY | Y | Y | N | Moderate |
| Kaffenberger et al. (2021) | N | Y | Y | N | Low |
| Ling et al. (2016) | N | Y | Y | N | Low |
| Meccariello et al. (2020) | PY | PY | PY | N | Moderate |
| More et al. (2013) | N | Y | Y | N | Low |
| Scott et al. (2021) | N | Y | Y | N | Low |
| Scott et al. (2023) | N | Y | Y | N | Low |
| Sharma et al. (2016) | N | Y | Y | N | Low |

**Table S10.**

**Assessment of Risk of Bias in Selection of the Reported Results (ROBINS-I Tool)**

**Legend:**

This table presents the assessment of **bias in selection of the reported results**, according to the **ROBINS-I tool**. The domain focuses on the potential for selective reporting of outcomes, analyses, or subgroups, which could affect the reliability of the findings.

**Response codes:**

- **Y** = Yes
- **N** = No
- **PY** = Probably Yes

An **Overall** judgement of **Low** or **Moderate** was assigned based on the potential for reporting bias.

|  | **Bias in selection of the reported results** | | | |
| --- | --- | --- | --- | --- |
| **Study (Year)** | 7.1 … multiple outcome *measurements* within the outcome domain? | 7.2 … multiple *analyses* of the intervention-outcome relationship? | 7.3 … different *subgroups*? | Overall |
| Amin et al. (2023) | N | PY | N | Moderate |
| Barbon et al. (2021) | N | N | N | Low |
| Hutcheson et al. (2019) | N | N | N | Low |
| Barbon et al. (2022) | N | N | N | Low |
| Chen et al. (2015) | N | N | N | Low |
| Dhanireddy et al. (2019) | N | N | N | Low |
| Genden et al. (2011) | N | N | N | Low |
| Hughes et al. (2023) | N | N | N | Low |
| Kaffenberger et al. (2021) | N | N | N | Low |
| Ling et al. (2016) | N | N | N | Low |
| Meccariello et al. (2020) | N | N | N | Low |
| More et al. (2013) | N | N | N | Low |
| Scott et al. (2021) | N | N | N | Low |
| Scott et al. (2023) | N | N | N | Low |
| Sharma et al. (2016) | N | N | N | Low |

**B. Randomised Controlled Trials (ROB-2 Tool)**

**Table S11.**

**Assessment of Risk of Bias Arising from the Randomisation Process (ROB-2 Tool)**

**Legend:**

This table presents the evaluation of **risk of bias due to the randomisation process**, using **Domain 1** of the **ROB-2 tool** for randomised controlled trials.

**Response codes:**

- **Y** = Yes
- **N** = No

The **overall judgement** for *Nichols et al. (2019)* was **Low**, indicating minimal risk of bias related to random sequence generation or allocation concealment.

| **Study (Year)** | **Domain 1: Risk of bias arising from the randomization process** | | | |
| --- | --- | --- | --- | --- |
|  | 1.1 Was the allocation sequence random? | 1.2 Was the allocation sequence concealed until participants were enrolled and assigned to interventions? | 1.3 Did baseline differences between intervention groups suggest a problem with the randomization process? | Overall |
| Nichols et al 2019 | Y | Y | N | Low |

**Table S12.**

**Assessment of Risk of Bias Due to Deviations from the Intended Interventions (Effect of Assignment to Intervention) (ROB-2 Tool)**

**Legend:**

This table presents the assessment of **risk of bias due to deviations from the intended interventions** (Domain 2) using the **ROB-2 tool** for randomised controlled trials. This domain evaluates the awareness of intervention assignment, occurrence and effect of deviations from protocol, and the appropriateness of analysis methods used.

**Response codes:**

- **Y** = Yes
- **N** = No
- **NA** = Not Applicable
- **PY** = Probably Yes
- **PN** = Probably No
- **NI** = No Information

The **overall judgement** of **Some concerns** reflects the potential for bias arising from lack of blinding or analytic shortcomings in estimating the effect of assignment to intervention.

| **Study (Year)** | **Domain 2: Risk of bias due to deviations from the intended interventions (effect of assignment to intervention)** | | | | | | | |
| --- | --- | --- | --- | --- | --- | --- | --- | --- |
|  | 2.1. Were participants aware of their assigned intervention during the trial? | 2.2. Were carers and people delivering the interventions aware of participants' assigned intervention during the trial? | 2.3. If Y/PY/NI to 2.1 or 2.2: Were there deviations from the intended intervention that arose because of the trial context? | 2.4 If Y/PY to 2.3: Were these deviations likely to have affected the outcome? | 2.5. If Y/PY/NI to 2.4: Were these deviations from intended intervention balanced between groups? | 2.6 Was an appropriate analysis used to estimate the effect of assignment to intervention? | 2.7 If N/PN/NI to 2.6: Was there potential for a substantial impact (on the result) of the failure to analyse participants in the group to which they were randomized? | Overall |
| Nichols et al 2019 | Y | Y | N | NA | NA | N | N | Some concerns |

**Table S13.**

**Assessment of Risk of Bias Due to Missing Outcome Data (ROB-2 Tool)**

**Legend:**

This table summarises the **risk of bias due to missing outcome data** for *Nichols et al. (2019)*, evaluated using **Domain 3** of the **ROB-2 tool**.

**Response codes:**

- **Y** = Yes
- **N** = No
- **PY** = Probably Yes
- **PN** = Probably No
- **NA** = Not Applicable

A judgement of **Low** indicates high confidence that missing data, if present, had little or no impact on the study’s outcome estimate.

| **Study (Year)** | **Domain 3: Risk of bias due to missing outcome data** | | | | |
| --- | --- | --- | --- | --- | --- |
|  | 3.1 Were data for this outcome available for all, or nearly all, participants randomized? | 3.2 If N/PN/NI to 3.1: Is there evidence that the result was not biased by missing outcome data? | 3.3 If N/PN to 3.2: Could missingness in the outcome depend on its true value? | 3.4 If Y/PY/NI to 3.3: Is it likely that missingness in the outcome depended on its true value? | Overall |
| Nichols et al 2019 | Y | NA | NA | NA | Low |

**Table S14.**

**Assessment of Risk of Bias in Measurement of the Outcome (ROB-2 Tool)**

**Legend:**

This table evaluates the **risk of bias in measurement of the outcome** for *Nichols et al. (2019)*, based on **Domain 4** of the **ROB-2 tool**.

**Response codes:**

- **Y** = Yes
- **N** = No
- **PY** = Probably Yes
- **PN** = Probably No
- **NI** = No Information

The overall judgement of **Some concerns** reflects potential risk due to assessor awareness, even though no direct influence on outcome measurement was deemed likely.

| **Study (Year)** | **Domain 4: Risk of bias in measurement of the outcome** | | | | | |
| --- | --- | --- | --- | --- | --- | --- |
|  | 4.1 Was the method of measuring the outcome inappropriate? | 4.2 Could measurement or ascertainment of the outcome have differed between intervention groups? | 4.3 If N/PN/NI to 4.1 and 4.2: Were outcome assessors aware of the intervention received by study participants? | 4.4 If Y/PY/NI to 4.3: Could assessment of the outcome have been influenced by knowledge of intervention received? | 4.5 If Y/PY/NI to 4.4: Is it likely that assessment of the outcome was influenced by knowledge of intervention received? | Overall |
| Nichols et al 2019 | N | N | Y | N | N | Some concerns |

**Table S15.**

**Assessment of Risk of Bias in Selection of the Reported Result (ROB-2 Tool)**

**Legend:**

This table presents the **risk of bias in selection of the reported result** for *Nichols et al. (2019)*, evaluated using **Domain 5** of the **ROB-2 tool**. This domain assesses whether selective reporting may have occurred due to flexible analytic strategies or outcome definitions.

**Response codes:**

- **Y** = Yes
- **N** = No
- **PY** = Probably Yes
- **PN** = Probably No
- **NI** = No Information

An **overall judgement** of **Low** indicates high confidence that the reported result was not influenced by selective reporting or post hoc analytic choices.

| **Study (Year)** | **Domain 5: Risk of bias in selection of the reported result** | | | |
| --- | --- | --- | --- | --- |
|  | 5.1 Were the data that produced this result analysed in accordance with a pre-specified analysis plan that was finalized before unblinded outcome data were available for analysis? | 5.2. ... multiple eligible outcome measurements (e.g. scales, definitions, time points) within the outcome domain? | 5.3 ... multiple eligible analyses of the data? | Overall |
| Nichols et al 2019 | Y | N | N | Low |

# **Section 3 – Extended GRADE Evidence Profiles**

**1. Extended GRADE Evidence Profiles**

**Table S16.**

**Extended GRADE Evidence Profile – Swallowing Function Assessed by MD Anderson Dysphagia Inventory (MDADI) at 3–6 or 6 Months Following Transoral Robotic Surgery (TORS) and Chemoradiotherapy (CRT) Treatments**

**Legend:**

This table summarises the **certainty of evidence** for swallowing function outcomes measured using the **MDADI** at approximately 3–6 months after **TORS** and **CRT**, based on the **GRADE approach**.

- **Risk of Bias**: Methodological quality of individual studies
- **Inconsistency**: Variability in results across studies
- **Indirectness**: Applicability of population, intervention, comparator, or outcome
- **Imprecision**: Degree of uncertainty due to sample size or confidence intervals
- **Publication Bias**: Suspected selective publication of results
- **Overall Certainty**: Final GRADE rating (High, Moderate, Low, or Very Low)

| **Author (Year)** | **Risk of Bias** | **Inconsistency** | **Indirectness** | **Imprecision** | **Publication Bias** | **Overall Certainty** |
| --- | --- | --- | --- | --- | --- | --- |
| **Barbon et al. (2021) / Hutcheson et al. (2019)** | Moderate | Low | No concerns | Low (moderate N and narrow CIs) | Unclear | **Moderate** |
| **Barbon et al. (2022)** | Low | Low | No concerns | Low (effect estimates are stable despite modest sample size) | Unclear | **High** |
| **More et al. (2013)** | Moderate | High (effect size deviates from other studies) | Moderate concerns (older methods) | High (very small N (20), no SDs reported) | Unclear | **Low** |

**Table S17.**

**Extended GRADE Evidence Profile – Swallowing Function Assessed by MD Anderson Dysphagia Inventory (MDADI) at 12 Months Following TORS and CRT Treatment**

**Legend:**

This **Extended GRADE evidence profile** summarises the certainty of evidence for **swallowing function at 12 months**, as measured by the **MDADI**, in studies comparing **TORS** and **CRT**.

- **Risk of Bias**: Assessed based on study design and reporting limitations
- **Inconsistency**: Evaluates variability in effect estimates across studies
- **Indirectness**: Assesses how directly the evidence applies to the review question
- **Imprecision**: Considers sample size, width of confidence intervals, and statistical significance
- **Publication Bias**: Suspected or detected selective reporting or missing studies

**Overall Certainty** is rated using GRADE standards: **High**, **Moderate**, or **Low**.

| **Author (Year)** | **Risk of Bias** | **Inconsistency** | **Indirectness** | **Imprecision** | **Publication Bias** | **Overall Certainty** |
| --- | --- | --- | --- | --- | --- | --- |
| **More et al. (2013)** | Moderate | High (effect size deviates from other studies) | Moderate (older methods) | High (very small sample (n=20), missing variance data) | Unclear | Low |
| **Nichols et al. (2019)** | Low | Low | No concerns | Moderate (decent sample (n=34), but CI includes null effect in statistical test) | Unclear | High |
| **Scott et al. (2021)** | Serious | Low | No concerns | Low (adequate sample (n=31), SDs reported, effect estimate stable) | Unclear | High |

**Table S18.**

**Extended GRADE Evidence Profile – Gastrostomy Tube Dependence at 3 Months**

**Legend:**

This table presents an **Extended GRADE evidence profile** for **gastrostomy tube dependence at 3 months** in patients treated with **TORS** or **CRT**.

**GRADE Domains:**

- **Risk of Bias**: Limitations in study methodology or reporting
- **Inconsistency**: Degree of variation across study results
- **Indirectness**: Applicability of evidence to the population and outcome of interest
- **Imprecision**: Statistical reliability, considering sample size and confidence intervals (CI)
- **Publication Bias**: Risk that relevant studies or results were not reported

**Overall Certainty** is assigned based on all domains, using the GRADE categories: **Very Low**, **Low**, **Moderate**, or **High**.

| **Author (Year)** | **Risk of Bias** | **Inconsistency** | **Indirectness** | **Imprecision** | **Publication Bias** | **Overall Certainty** |
| --- | --- | --- | --- | --- | --- | --- |
| **Dhanireddy et al. (2019)** | Low | High | No concerns | Serious (wide CI, not significant, n.r.) | Possible | Very Low |
| **Sharma et al. (2016)** | Moderate | None | No concerns | Minor (narrow CI, significant) | Unlikely | Moderate |

**Table S19.**

**Extended GRADE Evidence Profile – Gastrostomy Tube Dependence at 6 Months**

**Legend:**

This table presents an **Extended GRADE evidence profile** for **gastrostomy tube dependence at 6 months** in patients treated with **TORS** or **CRT**.

**GRADE Domains:**

- **Risk of Bias**: Study limitations that may influence results
- **Inconsistency**: Heterogeneity or variability across studies
- **Indirectness**: Relevance of study participants, interventions, or outcomes
- **Imprecision**: Level of certainty in effect estimates, considering confidence intervals (CI), sample size, and event rates
- **Publication Bias**: Risk that studies with unfavourable or null results were not published

The **Overall Certainty** reflects GRADE assessments, rated as **Very Low**, **Low**, **Moderate**, or **High**.

| **Author (Year)** | **Risk of Bias** | **Inconsistency** | **Indirectness** | **Imprecision** | **Publication Bias** | **Overall Certainty** |
| --- | --- | --- | --- | --- | --- | --- |
| **Amin et al. (2023)** | Moderate | Unclear | No concerns | Serious (CI spans null) | Possible | Low |
| **Dhanireddy et al. (2019)** | Low | High | No concerns | Serious (wide CI, not significant) | Possible | Very Low |
| **More et al. (2013)** | Moderate | None | No concerns | Minor (significant with narrow CI) | Unlikely | High |
| **Sharma et al. (2016)** | Moderate | Low | No concerns | Moderate (low event rate) | Unlikely | Moderate |

**Table S20.**

**Extended GRADE Evidence Profile – Gastrostomy Tube Dependence at 12 Months**

**Legend:**

This **Extended GRADE evidence profile** summarises the certainty of evidence for **gastrostomy tube dependence at 12 months** in patients treated with **TORS** or **CRT**, across seven studies.

**GRADE Domains:**

- **Risk of Bias**: Internal validity and design limitations
- **Inconsistency**: Differences in effect estimates between studies
- **Indirectness**: Applicability of evidence to the target population and outcome
- **Imprecision**: Reflected in wide or non-significant confidence intervals (CIs) or small sample sizes
- **Publication Bias**: Risk that evidence may be incomplete due to non-reporting or selective publication

The **Overall Certainty** is derived from domain ratings and categorised as **High**, **Moderate**, **Low**, or **Very Low**.

| **Author (Year)** | **Risk of Bias** | **Inconsistency** | **Indirectness** | **Imprecision** | **Publication Bias** | **Overall Certainty** |
| --- | --- | --- | --- | --- | --- | --- |
| **Amin et al. (2023)** | Moderate | Unclear | No concerns | Serious (n.r.) | Possible | Low |
| **Chen et al. (2015)** | Moderate | Some | No concerns | Serious (wide CI, not significant) | Possible | Low |
| **Genden et al. (2011)** | Serious | Some | No concerns | Very Serious (very wide CI, no significance) | Possible | Very Low |
| **Hughes et al. (2023)** | Low | None | No concerns | Minor (strong significance, narrow CI) | Unlikely | High |
| **More et al. (2013)** | Moderate | None | No concerns | Serious (wide CI despite strong RR) | Unlikely | Moderate |
| **Nichols et al. (2019)** | Low | Some | No concerns | Serious (wide CI, not significant) | Possible | Low |
| **Sharma et al. (2016)** | Low | Some | No concerns | Serious (non-significant, CI includes 1) | Possible | Low |

**Table S21.**

**Extended GRADE Evidence Profile – EORTC H&N35 Pain Scores Assessed at 12 Months**

**Legend:**

This table presents an **Extended GRADE evidence profile** for **pain scores** at 12 months, as assessed using the **EORTC H&N35** module in patients treated with **TORS** or **CRT**.

**GRADE Domains:**

- **Risk of Bias**: Design or reporting limitations
- **Inconsistency**: Variability across study results
- **Indirectness**: Applicability to the population, intervention, or outcome
- **Imprecision**: Reflected in high standard deviations (SDs), small effect sizes, or wide confidence intervals
- **Publication Bias**: Risk of missing or selectively reported findings

The **Overall Certainty** of the evidence is rated as **Low** or **Very Low**, based on domain-specific assessments.

| **Author (Year)** | **Risk of Bias** | **Inconsistency** | **Indirectness** | **Imprecision** | **Publication Bias** | **Overall Certainty** |
| --- | --- | --- | --- | --- | --- | --- |
| **Nichols et al. (2019)** | Low | High | No concerns | Serious (high SD, small difference) | Possible | Low |
| **Scott et al. (2021)** | Serious | High | No concerns | Serious (SDs large, small mean diff) | Possible | Very Low |

**2. Risk of Bias (RoB) Assessments**

**Table S22.**

**Risk of Bias Assessment Using the ROBINS-I Tool for Included Non-Randomised Controlled Studies**

**Legend:**

This table summarises the **risk of bias (RoB)** for included **non-randomised controlled studies** using the **ROBINS-I tool**, across seven domains:

- **Bias due to Confounding**
- **Selection Bias** (related to participant inclusion)
- **Bias in Classification of Interventions**
- **Bias due to Deviations from Intended Interventions**
- **Bias due to Missing Data**
- **Bias in Measurement of Outcomes**
- **Reporting Bias** (selective reporting)

Each domain is rated as **Low**, **Moderate**, or **Serious** risk. The **Overall RoB** reflects the highest concern in any domain, following ROBINS-I guidelines. Data for *Nichols et al. (2019)* were not available or not assessed and are marked with em dashes (—).

| **Study (Year)** | **Bias due to Confounding** | **Selection Bias** | **Bias in Classification** | **Bias due to Deviations from Intended Interventions** | **Bias due to Missing Data** | **Bias in Measurement** | **Reporting Bias** | **Overall RoB** |
| --- | --- | --- | --- | --- | --- | --- | --- | --- |
| Amin et al. (2023) | Moderate | Low | Moderate | Low | Low | Moderate | Moderate | Moderate |
| Barbon et al. (2021) ) / | Moderate | Moderate | Low | Low | Moderate | Moderate | Low | Moderate |
| Hutcheson et al. (2019) | Moderate | Moderate | Low | Low | Low | Moderate | Low | Moderate |
| Barbon et al. (2022) | Moderate | Low | Low | Low | Low | Low | Low | Low |
| Chen et al. (2015) | Moderate | Moderate | Low | Low | Moderate | Moderate | Low | Moderate |
| Dhanireddy et al. (2019) | Low | Low | Low | Low | Low | Low | Low | Low |
| Genden et al. (2011) | Serious | Moderate | Low | Low | Low | Moderate | Low | Serious |
| Hughes et al. (2023) | Moderate | Moderate | Low | Low | Moderate | Moderate | Low | Moderate |
| Kaffenberger et al. (2021) | Serious | Low | Low | Low | Moderate | Low | Low | Serious |
| Ling et al. (2016) | Low | Low | Low | Low | Low | Low | Low | Low |
| Meccariello et al. (2020) | Serious | Moderate | Low | Low | Moderate | Moderate | Low | Serious |
| More et al. (2013) | Moderate | Low | Low | Low | Low | Low | Low | Moderate |
| Nichols et al. (2019) | — | — | — | — | — | — | — | — |
| Scott et al. (2021) | Serious | Low | Low | Low | Moderate | Low | Low | Serious |
| Scott et al. (2023) | Serious | Low | Low | Low | Moderate | Low | Low | Serious |
| Sharma et al. (2016) | Moderate | Low | Low | Low | Low | Low | Low | Moderate |

**Table: Risk of Bias Assessment for Randomised Studies (RoB-2 Tool)**

**Table S23.**

**Risk of Bias Assessment Using the RoB-2 Tool for Included Randomised Controlled Trials**

**Legend:**

This table summarises the **risk of bias assessment** for the included **randomised controlled trial**, *Nichols et al. (2019)*, using the **RoB-2 tool**, which evaluates five domains:

- **Domain 1**: Randomisation process
- **Domain 2**: Deviations from intended interventions (effect of assignment)
- **Domain 3**: Missing outcome data
- **Domain 4**: Measurement of the outcome
- **Domain 5**: Selection of the reported result

Each domain is judged as **Low**, **Some concerns**, or **High** risk of bias. The **Overall** risk of bias reflects the collective judgement across all domains, following RoB-2 guidance.

| **Study (Year)** | **Domain 1: Risk of bias arising from the randomization process** | **Domain 2: Risk of bias due to deviations from the intended interventions (effect of assignment to intervention)** | **Domain 3: Risk of bias due to missing outcome data** | **Domain 4: Risk of bias in measurement of the outcome** | **Domain 5: Risk of bias in selection of the reported result** | **Overall** |
| --- | --- | --- | --- | --- | --- | --- |
| Nichols et al (2019) | Low | Some concerns | Low | Some concerns | Low | Some concerns |

**3. Inconsistency Analyses**

**Table S24.**

**Inconsistency Analysis of Swallowing Function Following TORS and CRT Treatments Assessed by MD Anderson Dysphagia Inventory (MDADI) at 3–6 and 12 Months Post-Intervention**

**Legend:**

This table summarises **MDADI scores** measuring swallowing function at **baseline**, **3–6 months**, and **12 months** post-treatment with **TORS (with or without adjuvant therapy)** versus **CRT**. Data include sample size (**N**), mean score, and standard deviation (**SD**). “n.r.” indicates data not reported.

Comparative inconsistencies across studies and timepoints inform GRADE assessments for **inconsistency** and support interpretation of clinical variability.

| **Study (Year)** | **TORS with or without Adjuvant Therapy** | | | **Control Treatment** | | |
| --- | --- | --- | --- | --- | --- | --- |
|  | **Number of Participants** | **Mean** | **Standard Deviation** | **Number of Participants** | **Mean** | **Standard Deviation** |
| **Baseline** |  |  |  |  |  |  |
| Barbon et al. (2021) / Hutcheson et al. (2019) | 75 | 90.4 | 11.2 | 182 | 92.3 | 8.9 |
| Barbon et al. (2022) | 38 | 92.0 | 8.7 | 97 | 93.5 | 8.4 |
| More et al. (2013) | 20 | 78 | n.r. | 20 | 78 | n.r. |
|  |  |  |  |  |  |  |
| **3-6 Months/ 6 Months** |  |  |  |  |  |  |
| Barbon et al. (2021) / Hutcheson et al. (2019) | 75 | 83.0 | 15.2 | 182 | 81.4 | 14.6 |
| Barbon et al. (2022) | 38 | 83.0 | 16.9 | 97 | 84.3 | 13.3 |
| More et al. (2013) | 20 | 76 | n.r. | 20 | 57 | n.r. |
|  |  |  |  |  |  |  |
| **12 Months** |  |  |  |  |  |  |
| More et al. (2013) | 20 | 78 | n.r. | 20 | 60 | n.r. |
| Nichols et al. (2019) | 34 | 80.2 | 13.1 | 34 | 86.7 | 1.4 |
| Scott et al. (2021) | 31 | 90.5 | 15.2 | 13 | 85 | 13.8 |


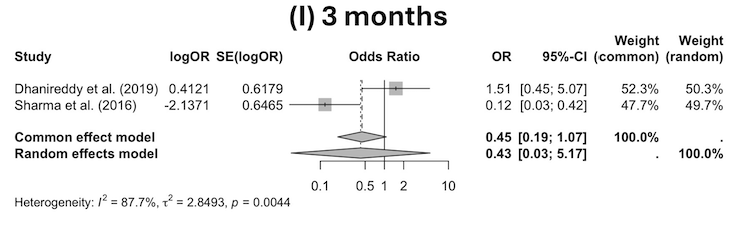


**Figure S5. Forest plot of odds ratios for gastrostomy tube dependence at 3 months post-treatment (TORS vs CRT).** Includes individual study estimates, pooled random-effects result, and heterogeneity statistics.

The forest plot presents a meta-analysis of two studies (Dhanireddy et al., 2019 and Sharma et al., 2016) evaluating outcomes at 3 months. The odds ratios (ORs) and confidence intervals (CIs) from the individual studies vary substantially, with Dhanireddy et al. reporting an OR of 1.51 (95% CI: 0.45 to 5.07), and Sharma et al. reporting an OR of 0.12 (95% CI: 0.03 to 0.42). This discrepancy is reflected in the pooled estimates.

Under the common effect (fixed-effect) model, the combined odds ratio is 0.45 (95% CI: 0.19 to 1.07), suggesting a non-significant trend toward reduced odds. However, due to significant between-study heterogeneity, the random effects model is more appropriate. The random effects pooled odds ratio is 0.43 (95% CI: 0.03 to 5.17), which remains statistically non-significant and shows a wider confidence interval, indicating greater uncertainty.

Heterogeneity Statistics:

- **I² = 87.7%**: This indicates that approximately 88% of the total variation across studies is due to heterogeneity rather than chance, suggesting substantial inconsistency between the study results.
- **τ² = 2.8493**: This represents the estimated between-study variance in the true effect sizes on the log odds ratio scale. A higher **τ**² suggests more variability in the underlying effect.
- **p = 0.0044**: This p-value for Cochran's Q test is statistically significant, indicating that the heterogeneity is unlikely to be due to chance alone.

Overall, there is substantial heterogeneity in the results of the included studies. While the pooled effect suggests a possible reduction in odds, the wide confidence intervals and significant heterogeneity limit the reliability of this estimate. Further exploration of the sources of heterogeneity is necessary prior to drawing definitive conclusions.


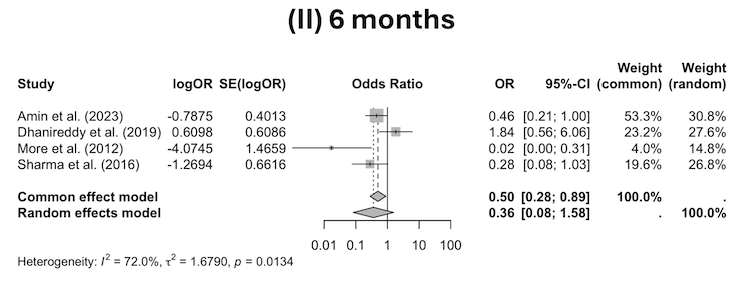


**Figure S6. Forest plot of odds ratios for gastrostomy tube dependence at 6 months (TORS vs CRT).** Displays study estimates, pooled random- and common-effect results, and heterogeneity statistics.

Study weights differ under common and random effects models, with **Amin et al. (2023)** contributing the most under both models. The diamond symbol represents the pooled effect size, while the squares reflect individual study estimates scaled by weight.

The forest plot summarises the results of a meta-analysis evaluating the effect of an intervention at 6 months, including four studies. The overall odds ratio (OR) under the common (fixed) effects model is **0.50 [95% CI: 0.28 to 0.89]**, suggesting a statistically significant reduction in the odds of the outcome in the intervention group. However, the random effects model, which accounts for between-study variability, yields an OR of **0.36 [95% CI: 0.08 to 1.58]**, indicating a wider confidence interval and lack of statistical significance.

Heterogeneity Statistics:

- **I² = 72.0%**: This indicates substantial heterogeneity, meaning that approximately 72% of the variability in effect sizes across studies is due to true differences rather than chance.
- **τ² = 1.6790**: This represents the estimated variance of the true effect sizes between studies. A higher τ² suggests greater heterogeneity.
- **p = 0.0134**: The p-value from the heterogeneity test (e.g., Cochran's Q test) is statistically significant (<0.05), confirming that the variation in effect sizes is unlikely to be due to random sampling error alone.

In conclusion, while the fixed effect model suggests a significant benefit of the intervention at 6 months, the presence of substantial heterogeneity and the non-significant result under the random effects model advise caution in interpretation and suggest variability in treatment effect across studies.


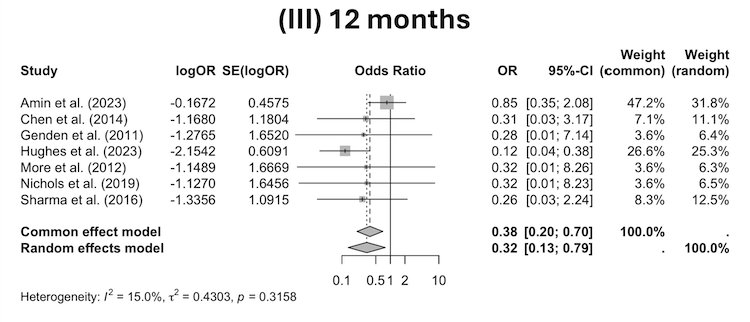


**Figure S7. Forest plot of odds ratios for gastrostomy tube dependence at 12 months (TORS vs CRT).** Shows individual study estimates, pooled random- and common-effect results, and heterogeneity statistics.

The forest plot presents the results of a meta-analysis assessing the effect of an intervention at **12 months**, based on seven studies. The pooled odds ratio (OR) using the **common (fixed) effects model** is **0.38 [95% CI: 0.20 to 0.70]**, and under the **random effects model**, the OR is **0.32 [95% CI: 0.13 to 0.79]**. Both models suggest a statistically significant reduction in the odds of the outcome at 12 months, favouring the intervention.

Heterogeneity Statistics:

- **I² = 15.0%**: This indicates low heterogeneity, suggesting that only 15% of the variability among the effect sizes is due to real differences across studies, with the remaining 85% attributed to random error.
- **τ² = 0.4303**: This value reflects a relatively small variance in the true effect sizes between studies.
- **p = 0.3158**: The non-significant p-value indicates that the null hypothesis of homogeneity cannot be rejected, suggesting that observed differences in study results are likely due to chance.

Overall, the findings indicate a consistent and statistically significant benefit of the intervention at 12 months. The low heterogeneity and significant pooled effect under both fixed and random effects models support the robustness and generalisability of the observed benefit.

***Pain Level Assessing under EORTC H&N 35 metrics at 12 Months***

**Table S25.**

**Imprecision Assessment: Gastrostomy Outcomes**

**Legend:**

This table summarises the **imprecision analysis** of gastrostomy-related outcomes at **12 months**, measured using the **EORTC H&N35** (European Organisation for Research and Treatment of Cancer Head & Neck module).

- Results are reported as **mean scores with standard deviations (SD)**.
- The magnitude of SDs relative to group means reflects substantial overlap and supports **GRADE downgrading for imprecision**, where applicable.

These findings are used in conjunction with effect estimates and confidence intervals in the GRADE framework to assess certainty in the evidence.

| **Author (Year)** | **Metric** | **Timescale** | **TORS ± Adjuvant therapy**  **Mean (SD)** | **Control Treatment**  **Mean (SD)** |
| --- | --- | --- | --- | --- |
| Nichols et al. (2019) | EORTC H&N 35 | 12 months | 13.3 (14.9) | 9.0 (12.4) |
| Scott et al. (2021) | EORTC H&N 35 | 12 months | 8.9 (13.8) | 11.1 (14.8) |

Two studies (Nichols et al., 2019; Scott et al., 2021) evaluated the effects of transoral robotic surgery (TORS) ± adjuvant radiotherapy on functional outcomes compared to control interventions. Both studies reported continuous outcome data (mean and standard deviation) on a comparable scale and involved similar patient populations and interventions, allowing for meta-analytic synthesis.

A random-effects meta-analysis was performed using the DerSimonian–Laird method. The pooled analysis showed conflicting directions of effect between studies: Nichols et al. (2019) reported a mean difference (MD) favouring TORS (MD = −4.3), while Scott et al. (2021) reported a mean difference favouring the control group (MD = +2.2). The combined I² statistic was 22.4%, indicating low statistical heterogeneity. The between-study variance (τ²) was estimated at 4.74.

The 95% confidence intervals for each study overlapped zero, and the pooled estimates also included the null value, suggesting no statistically significant overall effect. Confidence intervals for the individual studies were Nichols et al. (−10.82 to 2.22) and Scott et al. (−6.93 to 11.33), reflecting substantial imprecision.

Based on the GRADE framework, the overall certainty of evidence was rated as **low**, due to serious concerns about imprecision, despite low inconsistency and acceptable indirectness. Risk of bias and publication bias could not be fully assessed with the available data.

**4. Publication Bias Assessments**

***Gastrostomy Tube Dependence at 3 Months***

A comprehensive evaluation of publication bias was conducted for the two studies reporting 3-month gastrostomy tube dependence following transoral robotic surgery (TORS), with or without adjuvant therapy, compared to control interventions.

**Statistical Assessment:**
Given the limited number of studies (n = 2), formal statistical tests for publication bias—namely Egger’s regression test and Begg’s rank correlation test—were not feasible, as these methods require a minimum of 3–5 studies to produce valid and interpretable results. Similarly, a trim-and-fill analysis was not performed due to insufficient data.

**Study Characteristics and Effect Estimates:**
Marked heterogeneity was observed in the reported outcomes. Dhanireddy et al. (2019) reported a non-significant effect favouring the control group (OR = 1.51; 95% CI: 0.443–5.15; p = 0.51), while Sharma et al. (2016) demonstrated a statistically significant benefit for TORS (OR = 0.115; 95% CI: 0.0329–0.411; p < 0.001).

**Funnel Plot Evaluation:**


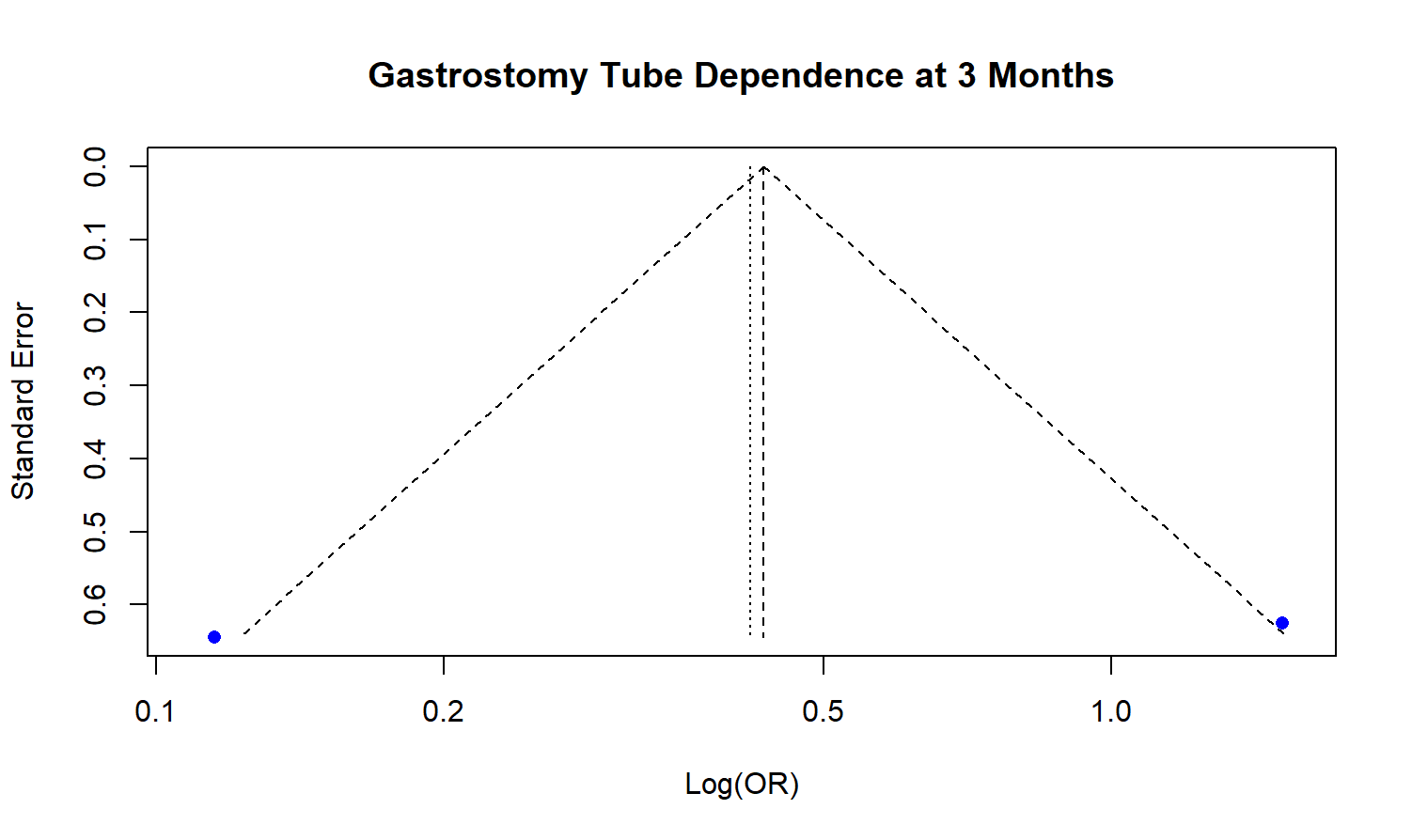


**Figure S8. Funnel plot of gastrostomy tube dependence at 3 months (TORS vs CRT).** Displays study log[OR] vs standard error; dashed line shows pooled effect; triangle indicates expected unbiased range.

Given the limited data points, formal statistical tests (e.g. Egger’s test) were not performed.

Visual inspection of the funnel plot revealed asymmetry, with only two widely divergent effect estimates and no intermediate values. Such a distribution raises concerns regarding selective reporting or study-level heterogeneity but precludes meaningful interpretation due to the small sample size.

**Heterogeneity Considerations:**
The observed discrepancies may reflect substantial clinical and methodological heterogeneity. Clinical differences included variation in patient selection, adjuvant therapy protocols, and surgical techniques. Methodologically, inconsistencies in outcome assessment, follow-up duration, and potential centre-specific effects were evident.

**Limitations:**
The limited number of studies and the high degree of heterogeneity restricted the ability to assess publication bias quantitatively. As such, while visual indicators suggest potential bias or true clinical variation, definitive conclusions cannot be drawn.

***Gastrostomy Tube Dependence at 6 Months***

An assessment of publication bias was performed across four studies evaluating gastrostomy tube dependence at 6 months post-treatment.

**Statistical Tests:**
Egger’s regression test yielded an intercept of -1.79 (95% CI: -7.23 to 3.66; p = 0.34), and Begg’s test showed Kendall’s τ = -0.33 (p = 0.50), indicating no statistically significant evidence of small-study effects or publication bias.

**Trim-and-Fill Analysis:**
The trim-and-fill method estimated zero missing studies, and the adjusted effect size (logOR = -1.69) was consistent with the original findings. This suggests robustness of the observed effect, despite mild funnel plot asymmetry.

**Funnel Plot Evaluation:**


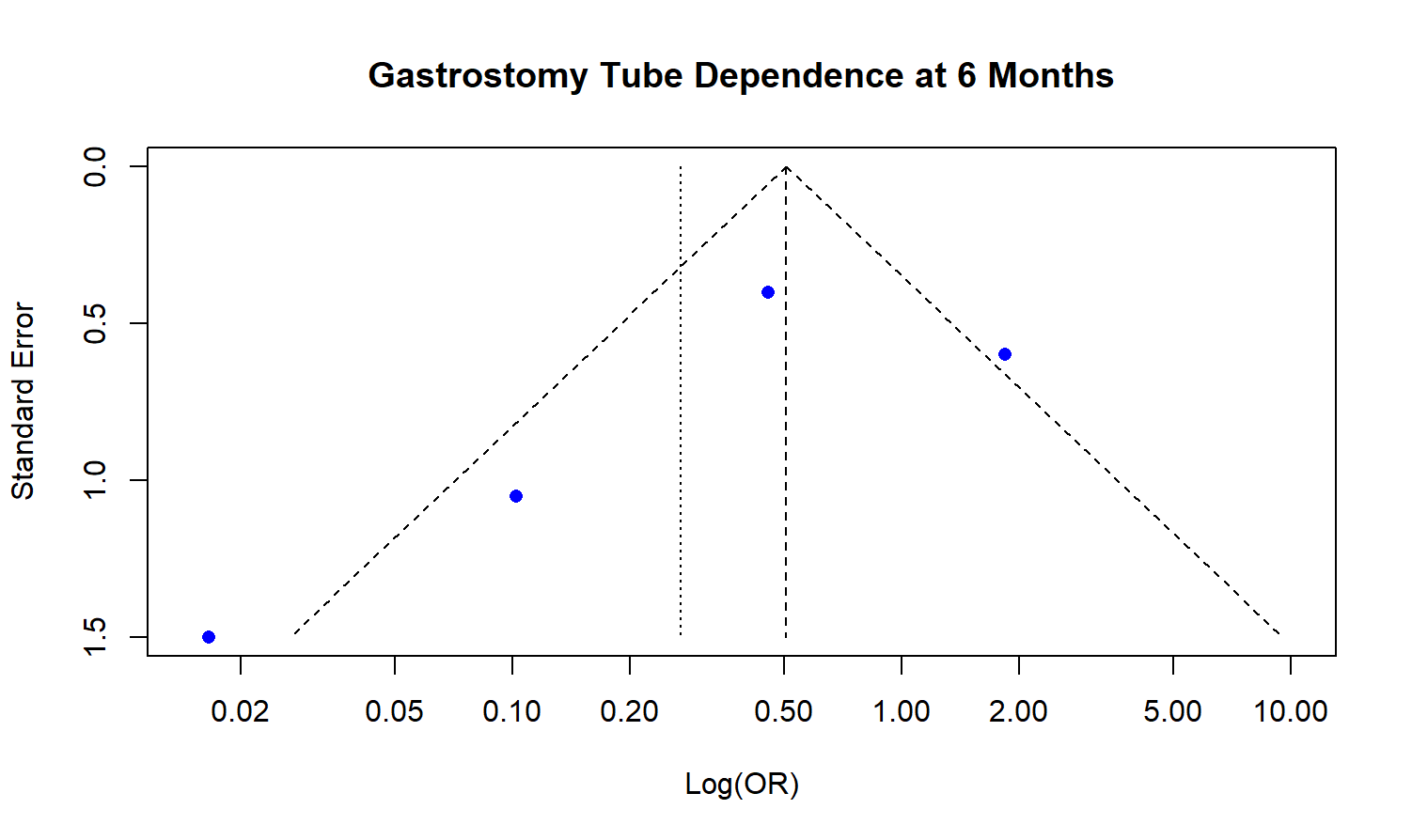


**Figure S9. Funnel plot of gastrostomy tube dependence at 6 months (TORS vs CRT).** Displays log[OR] vs standard error; dashed line shows pooled effect; asymmetry suggests possible publication bias.

This plot contributes to the **GRADE assessment of publication bias** for this outcome.

Visual inspection indicated mild asymmetry, with three studies favouring TORS clustered on the left and one study (Dhanireddy et al., 2019) favouring the control group on the right. However, the asymmetry was not statistically significant and may reflect genuine heterogeneity.

**Limitations:**
The small number of studies (n = 4) limits the sensitivity of statistical methods used to detect bias. Additionally, the reliability of the trim-and-fill method is diminished when fewer than 10 studies are included. Therefore, findings should be interpreted with caution.

**Conclusion:**
While visual inspection suggested asymmetry, statistical tests did not confirm the presence of publication bias. The observed variation may reflect true clinical differences rather than selective reporting. Nonetheless, the potential for undetected bias remains due to the small sample size.

***Gastrostomy Tube Dependence at 12 Months***

Publication bias was assessed across seven studies reporting 12-month gastrostomy tube dependence outcomes.

**Statistical Tests:**
Egger’s test showed no significant small-study effects (intercept = -0.79; 95% CI: -2.91 to 1.33; p = 0.40), and Begg’s test indicated no significant rank correlation (Kendall’s τ = -0.14; p = 0.71).

**Funnel Plot Evaluation:**


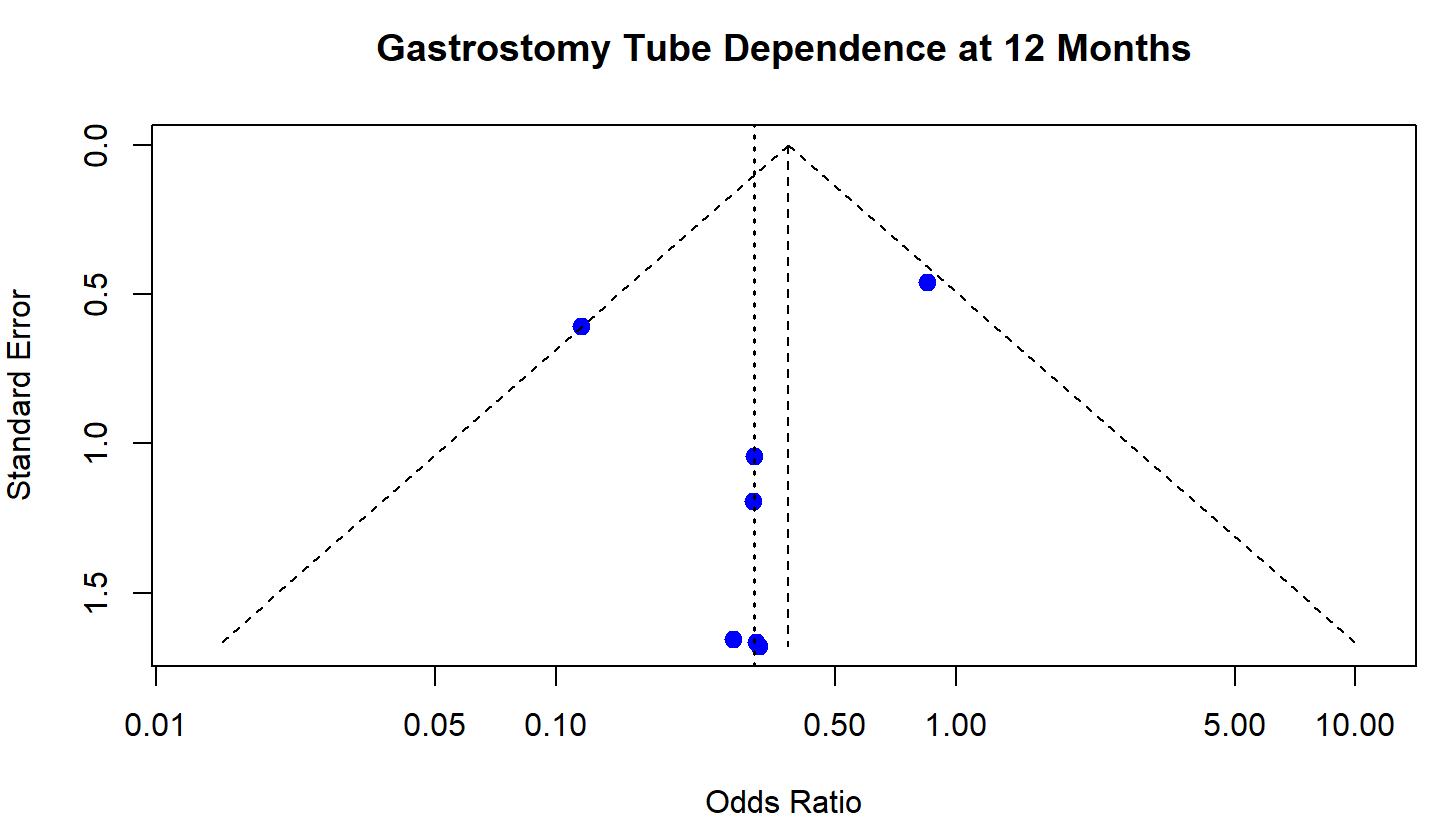


**Figure S10. Funnel plot of gastrostomy tube dependence at 12 months (TORS vs CRT).** Displays OR vs standard error; dashed line shows pooled effect; symmetry suggests low publication bias risk.

The funnel plot demonstrated a symmetrical distribution of effect estimates around the pooled value, with no substantial gaps suggesting missing studies.

**Conclusion:**
Neither statistical tests nor visual inspection provided evidence of publication bias. However, the number of included studies (n = 7) was still below the threshold typically required for robust application of certain bias detection methods, such as the trim-and-fill approach, which was therefore not conducted.

***MDADI Scores at 12 Months***

Publication bias was assessed across two studies reporting 12-month MDADI scores.

**Statistical Tests:**
Given the limited number of studies (n = 2), formal statistical tests for publication bias—namely Egger’s regression test and Begg’s rank correlation test—were not feasible, as these methods require a minimum of 3–5 studies to produce valid and interpretable results. Similarly, a trim-and-fill analysis was not performed due to insufficient data.

**Study Characteristics and Effect Estimates:**
Marked heterogeneity was observed in the reported outcomes. Nichols et al. (2019) reported a significant effect favouring the control group (Effect estimate 0.553, 95% CI: 0.029-1.076, p=0.04), while Scott et al. (2021) demonstrated a statistically non-significant benefit for TORS (-0.401 95% CI: -1.000-0.198, p=0.006).

**Funnel Plot Evaluation:**


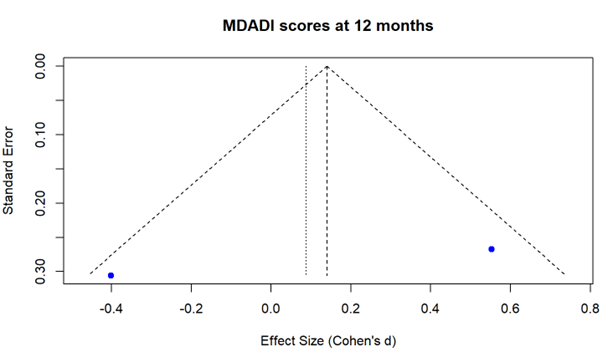


**Figure S11. Funnel plot of MDADI swallowing scores at 12 months (TORS vs CRT).** Displays effect size vs standard error; dashed line shows pooled estimate; limited by small number of studies.

Visual inspection of the funnel plot revealed asymmetry, with only two widely divergent effect estimates and no intermediate values. Such a distribution raises concerns regarding selective reporting or study-level heterogeneity but precludes meaningful interpretation due to the small sample size.

**5. Imprecision Calculations**

- **Minimal Important Differences (MIDs)** used for downgrading.

If 95% CI of effect size includes no difference and carries strong absolute risk reduction (larger than or equal to 0.05), the study has to be downgraded due to imprecision.

**Table S26.**

**Summary of Effect Estimates for Gastrostomy Tube Dependence Following TORS and CRT**

**Legend:**

This table summarises **odds ratios (ORs)**, **confidence intervals (CIs)**, and **risk reductions** for **gastrostomy tube dependence** at multiple time points after treatment with **TORS** or **CRT**.

- **Absolute Risk Reduction (ARR)**: Proportion of patients who benefit from TORS compared to CRT.
- **Relative Risk Reduction (RRR)**: Proportional reduction in risk relative to the control group.
- **Significance**: Based on reported *p*-values.
- **Downgrade**: Indicates that the study may have contributed to lower GRADE certainty due to imprecision or inconsistency.

| **Author (Year)** | **Time Scale** | **Odd Ratio** | **Estimated 95% Confidence Interval** | **Significance** | **Absolute Risk Reduction** | **Relative Risk Reduction** | **Comment** |
| --- | --- | --- | --- | --- | --- | --- | --- |
| Dhanireddy et al. (2019) | at 3 months | 1.51 | [0.443, 5.15] | 0.51 | -0.0300 | -0.108 |  |
| Sharma et al. (2016) | at 3 months | 0.115 | [0.033, 0.411] | <0.001 | 0.344 | 0.817 |  |
| Amin et al. (2023) | at 6 months | 0.455 | [0.207, 0.998] | 0.04 | 0.11 | 0.444 |  |
| Dhanireddy et al. (2019) | at 6 months | 1.84 | [0.570, 5.93] | 0.31 | -0.0714 | -0.231 |  |
| More et al. (2013) | at 6 months | 0 | [0.000885, 0.314] | <0.0001 | 0.600 | 1.00 |  |
| Sharma et al. (2016) | at 6 months | 0.102 | [0.013, 0.795] | 0.002 | 0.205 | 0.875 |  |
| Amin et al. (2023) | at 12 months | 0.846 | [0.346, 2.079] | 0.71 | 0.019 | 0.0864 | Downgrade |
| Chen et al. (2015) | at 12 months | 0.311 | [0.0302, 3.25] | 0.499 | 0.0645 | 0.667 | Downgrade |
| Genden et al. (2011) | at 12 months | 0.278 | [0.0108, 7.12] | 1.00 | 0.0385 | 1.00 |  |
| Hughes et al. (2023) | at 12 months | 0.116 | [0.0354, 0.380] | <0.001 | 0.200 | 0.852 |  |
| More et al. (2013) | at 12 months | 0.317 | [0.0122, 8.28] | 1.00 | 0.0500 | 1.00 | Downgrade |
| Nichols et al. (2019) | at 12 months | 0.323 | [0.0121, 8.67] | 1.00 | 0.0294 | 1.00 |  |
| Sharma et al. (2016) | at 12 months | 0.313 | [0.0368, 2.19] | 0.108 | 0.0539 | 0.678 | Downgrade |

**6. Absolute Effect Estimates**

**MDADI Swallowing Metrics**

***Swallowing Functions TORS and CRT Treatments under MD Anderson Dysphagia Inventory 3-6/ 6 Months after Intervention***

A total of three studies were included in the meta-analysis, encompassing both TORS and control treatment groups. Paired mean differences in MDADI scores from baseline to 6 months post-treatment were extracted or calculated. The pooled mean change for the TORS group was –7.05 points, indicating a moderate decline in swallowing-related quality of life. In comparison, the control group showed a larger pooled mean decrease of –11.02 points over the same period.

To further quantify the magnitude of these changes, effect sizes (Cohen’s d) were computed and pooled using a fixed-effect model. The TORS group demonstrated a pooled Cohen’s d of –0.61 (95% CI approximately –0.79 to –0.42), reflecting a moderate negative effect. The control group had a larger pooled Cohen’s d of –0.86 (95% CI approximately –1.01 to –0.72), indicating a more pronounced deterioration in swallowing function.

These findings suggest that although both treatment groups experienced statistically significant declines in MDADI scores at 6 months, the magnitude of decline was consistently greater in the control group. This supports the possibility that TORS may be associated with a more favourable swallowing outcome relative to alternative treatments.

To assess the statistical significance of differences in swallowing-related quality of life between TORS and control groups, independent-sample t-tests were performed on the mean change in MDADI scores between baseline and 3–6 months. Only studies with complete standard deviation data were included in this analysis.

- For **Barbon et al. (2021)**, the difference in mean change between the TORS and control groups approached but did not reach statistical significance (t = 1.70, p = 0.091).
- For **Barbon et al. (2022)**, the difference was not statistically significant (t = 0.07, p = 0.948).

While Barbon et al. (2021) showed a modest improvement in swallowing outcomes favouring the TORS group, neither study demonstrated a statistically significant difference at the conventional α = 0.05 level. These findings suggest that, despite potential clinical relevance, the observed differences may be influenced by variability in sample sizes and standard deviations.

***Swallowing Functions TORS and CRT Treatments under MD Anderson Dysphagia Inventory 12 Months after Intervention***

Three studies were included in the meta-analysis, comparing swallowing-related quality of life outcomes between patients treated with TORS with or without adjuvant therapy and those receiving control treatments. Changes in MDADI scores from baseline to 12 months were analysed, and standardised mean differences (Cohen’s d) were computed to assess the magnitude of change within each group.

In the TORS group, the pooled effect size was **Cohen’s d = –0.63 (95% CI: –1.21 to –0.06)**, indicating a moderate decline in swallowing-related quality of life over 12 months. In contrast, the control group demonstrated a more substantial decline, with a pooled effect size of **Cohen’s d = –1.68 (95% CI: –2.79 to –0.57)**. This large negative effect suggests a more pronounced deterioration in swallowing function among patients receiving non-TORS treatments.

The direction and magnitude of the effect sizes were consistent across studies, with all included studies showing greater functional decline in the control group relative to the TORS group. These results support the hypothesis that TORS may be associated with better preservation of swallowing-related quality of life compared to traditional or non-robotic treatment approaches.

To assess the statistical significance of differences in swallowing-related quality of life between TORS and control groups, independent-sample t-tests were performed on the mean change in MDADI scores over 12 months. Only studies with complete standard deviation data were included in this analysis.

- For **Nichols et al. (2019)**, the difference in mean change between the TORS and control groups approached but did not reach statistical significance (t = –1.67, p = 0.103).
- For **Scott et al. (2021)**, the difference was not statistically significant (t = 0.35, p = 0.726).

While both studies demonstrated clinically meaningful differences in effect sizes favouring the TORS group, these differences were not statistically significant at the conventional α = 0.05 level. This may be attributable to variability in sample sizes and within-group standard deviations.

**Gastrostomy Tube Dependence**

**Study Design**

This is a retrospective, observational study aimed at assessing the efficacy of TORS **±** Adjuvant therapy compared to the control group in preventing specified events. The data were analysed using statistical methods including calculation of odds ratios, confidence intervals, and significance levels, in addition to assessing the absolute risk reduction and number needed to treat.

**Data Collection**

Data were collected from two groups: **TORS ± Adjuvant** and **CRT**. Each group was assessed for the number of events (defined as a specific clinical outcome) and the number of non-events (absence of the clinical outcome). The data are summarised in the following 2x2 contingency table:

**Table S27.**

**Example Data for Bias Domain Judgements**

**Legend:**

This table presents a numerical example used to support **bias domain judgements**, such as for **selective outcome reporting** or **baseline imbalances**, during **risk of bias assessment** (e.g. ROBINS-I or RoB-2).

- **Events** typically refer to the outcome of interest (e.g. gastrostomy tube dependence).
- The **TORS + Adjuvant** group includes participants who received TORS followed by radiotherapy or chemoradiotherapy.
- The **Control** group received CRT alone.

| **Group** | **Events** | **No Events** | **Total** |
| --- | --- | --- | --- |
| **TORS ± Adjuvant** | 3 | 36 | 39 |
| **CRT** | 37 | 51 | 88 |

**Statistical Analysis**

- **Odds Ratio (OR)**: The odds ratio was calculated using the formula:

$$OR= \frac{(a\times d)}{(b\times c)}$$

where:

- *a* is the number of events in the **TORS ± Adjuvant** group,
- *b* is the number of non-events in the **TORS ± Adjuvant** group,
- *c* is the number of events in the **Control** group,
- *d* is the number of non-events in the **Control** group.

If the TORS + Adjuvant or Control group has no events, we applied a **continuity correction** of 0.5 to each cell of the table to avoid division by zero.

- **95% Confidence Interval (CI)**: The 95% CI for the odds ratio was estimated using the log-transformation method. The standard error (SE) of the log-odds ratio was calculated as:

$$SE= \sqrt{\frac{1}{a}+\frac{1}{b}+\frac{1}{c}+\frac{1}{d}}$$

The lower and upper limits of the confidence interval were then derived by exponentiating the log-odds ratio ± 1.96 times the standard error.

- **Significance Level (p-value)**: The p-value was calculated using Fisher's Exact Test for a 2x2 table. This non-parametric test is appropriate for small sample sizes and determines whether the observed distribution of events across the two groups is statistically significant.
- **Absolute Risk Reduction (ARR)**: The absolute risk reduction was calculated as the difference in event rates between the **Control** and **TORS ± Adjuvant** groups:

$$ARR=Event Rate (Control)\mathbf{-}Event Rate (TORS\boldsymbol{\pm}Adjuvant)$$

Where the event rate is the proportion of events in each group, calculated as:

$$Event Rate= \frac{Number of Events}{Total Number of Participants}$$

​

- **Relative Risk Reduction (RRR)**: The relative risk reduction represents the proportion by which the risk is reduced in the treatment group compared to the control group. It is calculated as the difference between the event rates in the control and treatment groups, divided by the event rate in the control group:

$$RRR= \frac{Event Rate (Control)-Event Rate (TORS\boldsymbol{\pm}Adjuvant)}{Event Rate (Control)}$$

- **Number Needed to Treat (NNT)**: The number needed to treat was calculated by taking the inverse of the absolute risk reduction:

**Table S28.**

**Effect Estimates and Clinical Interpretation of Gastrostomy Tube Dependence Following TORS and CRT**

**Legend:**

- **n.r.** = Not Reported
- **ARR** = Absolute Risk Reduction
- **RRR** = Relative Risk Reduction
- **NNT/NNH** = Number Needed to Treat / Number Needed to Harm
  - Positive values = **NNT** (benefit from TORS)
  - Negative values = **NNH** (higher risk with TORS)

This table provides a comparative overview of **effect size estimates** and their clinical implications for **gastrostomy tube dependence** following **TORS** vs **CRT**, including interpretation aids like NNT and RRR to inform **GRADE certainty judgements**.

|  |  | **TORS ± Adjuvant therapy** | | **Control Treatment** | |  |  |  |  |  |  |
| --- | --- | --- | --- | --- | --- | --- | --- | --- | --- | --- | --- |
| **Author (Year)** | **Time Scale** | **Number** | **Percentage (%)** | **Number** | **Percentage (%)** | **Odd Ratio** | **Estimated 95% Confidence Interval** | **Significance** | **Absolute Risk Reduction** | **Relative Risk Reduction** | **Number Need to Treat/ Number Needed to Harm** |
| Dhanireddy et al. (2019) | at 3 months | n.r. | n.r. | n.r. | n.r. | 1.51 | [0.443, 5.15] | 0.51 | -0.0300 | -0.108 | 34 |
| Sharma et al. (2016) | at 3 months | 3 | 7.69 | 37 | 42.0 | 0.115 | [0.033, 0.411] | <0.001 | 0.344 | 0.817 | 3 |
| Amin et al. (2023) | at 6 months | n.r. | n.r. | n.r. | n.r. | 0.455 | [0.207, 0.998] | 0.04 | 0.11 | 0.444 | 10 |
| Dhanireddy et al. (2019) | at 6 months | n.r. | n.r. | n.r. | n.r. | 1.84 | [0.570, 5.93] | 0.31 | -0.0714 | -0.231 | 14 |
| More et al. (2013) | at 6 months | 0 | 0 | 12 | 60.0 | 0 | [0.000885, 0.314] | <0.0001 | 0.600 | 1.00 | 2 |
| Sharma et al. (2016) | at 6 months | 1 | 2.56 | 18 | 20.5 | 0.102 | [0.013, 0.795] | 0.002 | 0.205 | 0.875 | 6 |
| Amin et al. (2023) | at 12 months | n.r. | n.r. | n.r. | n.r. | 0.846 | [0.346, 2.079] | 0.71 | 0.019 | 0.0864 | 53 |
| Chen et al. (2015) | at 12 months | 1 | 3.23 | 3 | 9.68 | 0.311 | [0.0302, 3.25] | 0.499 | 0.0645 | 0.667 | 16 |
| Genden et al. (2011) | at 12 months | 0 | 0 | 1 | 3.85 | 0.278 | [0.0108, 7.12] | 1.00 | 0.0385 | 1.00 | 26 |
| Hughes et al. (2023) | at 12 months | 4 | 3.5 | 12 | 23.5 | 0.116 | [0.0354, 0.380] | <0.001 | 0.200 | 0.852 | 5 |
| More et al. (2013) | at 12 months | 0 | 0 | 1 | 5 | 0.317 | [0.0122, 8.28] | 1.00 | 0.0500 | 1.00 | 20 |
| Nichols et al. (2019) | at 12 months | 0 | 0 | 1 | 2.94 | 0.323 | [0.0121, 8.67] | 1.00 | 0.0294 | 1.00 | 34 |
| Sharma et al. (2016) | at 12 months | 1 | 3 | 7 | 11 | 0.313 | [0.0368, 2.19] | 0.108 | 0.0539 | 0.678 | 19 |

# **Section 4 – Comparative Analysis of Swallowing Function Outcomes**

These tables present a comparative analysis of swallowing function outcomes between patients treated with TORS and those who received other treatments such as radiotherapy (RT) or CRT, using data from multiple studies. Table S29 focuses on the MDADI, a patient-reported measure of swallowing-related quality of life. Most studies reported no significant difference in scores between TORS and control groups, though some (e.g., Nichols et al., More et al.) found statistically significant benefits for TORS at specific time points. This suggests that while TORS may offer swallowing-related quality-of-life advantages in some cases, the overall trend is mixed and dependent on time post-treatment and study design.

**Table S29.**

**MD Anderson Dysphagia Inventory (MDADI) Swallowing Function Scores for TORS vs. Control Groups Across Studies and Time Points**

**Legend:**

This table summarises **MDADI scores** (higher = better swallowing function) for patients undergoing **TORS** versus **control treatments (primarily CRT or radiotherapy subgroups)** at various time points.

- **p-values** reflect between-group comparisons.
- **n.r.** = not reported.
- Statistical significance is noted where explicitly reported or inferable from data.

This table supports **GRADE assessments** of **inconsistency**, **imprecision**, and **effect size** in swallowing function outcomes across studies.

| **Author (Year)** | **Time Point** | **TORS Group Score** | **Control Group Score** | **p-value** | **Notes** |
| --- | --- | --- | --- | --- | --- |
| Barbon et al. (2022) | Baseline | 92 | Unilateral RT: 92.9 / Bilateral RT: 93.2 | 0.9 |  |
| Barbon et al. (2022) | 3–6 months | 83 | Unilateral RT: 88.0 / Bilateral RT: 82.0 | 0.38 |  |
| Barbon et al. (2022) | 24 months | 85.5 | Unilateral RT: 86.4 | 0.99 |  |
| Nichols et al. (2019) | 12 months | 80.1 | 86.9 | 0.04 | Statistically significant |
| Scott et al. (2021) | Baseline | 93.3 | 90 | n.r. |  |
| Scott et al. (2021) | 12 months | 90.5 | 85 | n.r. |  |
| More et al. (2013) | 3 months | 62 | 56 | Insignificant |  |
| More et al. (2013) | 6 months | 76 | 57 | 0.004 | Statistically significant |
| More et al. (2013) | 12 months | 78 | 60 | 0.006 | Statistically significant |

Tables S30 and S31 present more objective and functional data. Table S30 summarises outcomes from the Dynamic Imaging Grade of Swallowing Toxicity (DIGEST) and Modified Barium Swallow Impairment Profile (MBSImP). Significant improvements were noted in certain MBSImP parameters (e.g., laryngeal vestibule closure and pharyngeal contraction) favouring TORS. Table S31 uses the Functional Oral Intake Scale (FOIS) to assess swallowing ability, with early post-treatment advantages for TORS and significant score distribution differences favouring TORS in longer-term follow-up (Hughes et al.). Collectively, the data indicate that TORS may offer potentially lasting advantages in swallowing function compared to traditional treatments, though findings vary across measures and time points.

**Table S30.**

**Dynamic Imaging Grade of Swallowing Toxicity (DIGEST) Swallowing Function Scores for TORS vs. Control Groups Across Studies and Time Points**

**Legend:**

This table presents swallowing function outcomes measured by the **DIGEST scale** and **MBSImP components** in patients undergoing **TORS** versus **CRT** or other control treatments across various time points.

- **DIGEST** = Dynamic Imaging Grade of Swallowing Toxicity
- **MBSImP** = Modified Barium Swallow Impairment Profile
- **p-values** denote statistical significance between groups; **n.r.** = not reported.

Results contribute to **GRADE assessments** regarding **inconsistency**, **indirectness**, and **precision** in functional swallowing outcomes.

| **Author (Year)** | **Time Point** | **Metric** | **TORS Group Score** | **Control Group Score** | **p-value** | **Notes** |
| --- | --- | --- | --- | --- | --- | --- |
| Barbon et al. (2021) | Baseline | Dynamic Imaging Grade of Swallowing Toxicity (DIGEST) | 25 | 16 | 0.06 |  |
| Barbon et al. (2021) | 3–6 months | DIGEST | 45 | 42 | 0.93 |  |
| Barbon et al. (2021) | 3–6 months | MBSImP - Laryngeal Vestibule Closure | 27% | 41% | 0.02 | Significant |
| Barbon et al. (2021) | 3–6 months | MBSImP - Pharyngeal Contraction | 62% | 53% | 0.001 | Significant |
| Scott et al. (2021) | 3 months | Proportion with Grade 1 or 2 DIGEST | 54.8 | 23.1 | n.r. |  |
| Scott et al. (2021) | 12 months | Proportion with Grade 1 or 2 DIGEST | 29.0% | 45.5% | n.r. |  |
| Scott et al. (2023) | 1 year | Proportion with Grade 1 or 2 DIGEST | 29 | 45.5 | n.r. |  |
| Scott et al. (2023) | 3 years | Proportion with Grade 1 or 2 DIGEST | 11.5 | 9.09 | n.r. |  |

**Table S30.**

**Dynamic Imaging Grade of Swallowing Toxicity (DIGEST) Swallowing Function Scores for TORS vs. Control Groups Across Studies and Time Points**

**Legend:**

This table presents swallowing function outcomes measured by the **DIGEST** scale and **MBSImP** components in patients undergoing **TORS** versus **CRT** or other control treatments across various time points.

- **DIGEST** = *Dynamic Imaging Grade of Swallowing Toxicity*
- **MBSImP** = *Modified Barium Swallow Impairment Profile*
- *p-values* denote statistical significance between groups
- **n.r.** = *not reported*.

Results contribute to **GRADE assessments** regarding **inconsistency**, **indirectness**, and **precision** in functional swallowing outcomes.

| **Author (Year)** | **Time Point** | **TORS Group Score** | **Control Group Score** | **p-value** | **Notes** |
| --- | --- | --- | --- | --- | --- |
| Genden et al. (2011) | 2 weeks | 5.5 ± 0.2 | 3.3 ± 0.6 | <0.001 | Significant |
| Genden et al. (2011) | 3 months | n.r. | n.r. | Insignificant |  |
| Genden et al. (2011) | 6 months | n.r. | n.r. | Insignificant |  |
| Genden et al. (2011) | 9 months | n.r. | n.r. | Insignificant |  |
| Genden et al. (2011) | 12 months | n.r. | n.r. | Insignificant |  |
| Hughes et al. (2023) | 1 year | -6: 1.0 | -6: 1.0 | 0.008 | Distribution of score changes |
| Hughes et al. (2023) | 1 year | -5: 1.0 | -5: 1.0 | 0.008 |  |
| Hughes et al. (2023) | 1 year | -4: 1.0 | -4: 2.0 | 0.008 |  |
| Hughes et al. (2023) | 1 year | -3: 1.0 | -3: 2.0 | 0.008 |  |
| Hughes et al. (2023) | 1 year | -2: 2.9 | -2: 10.0 | 0.008 |  |
| Hughes et al. (2023) | 1 year | -1: 29.4 | -1: 24.0 | 0.008 |  |
| Hughes et al. (2023) | 1 year | 0: 58.8 | 0: 34.8 | 0.008 |  |
| Hughes et al. (2023) | 1 year | +1: 4.9 | +1: 19.2 | 0.008 |  |
| Hughes et al. (2023) | 1 year | +2: 0 | +2: 2.2 | 0.008 |  |
