# Supplementary material for: A Meta‐analysis of Functional Outcomes and Recovery Metrics Comparing Transoral Robotic Surgery and (Chemo)Radiotherapy
Source: Otolaryngol Head Neck Surg. 2025 Dec 7;174(1):57–76. doi: 10.1002/ohn.70069 (PMC12794744; doi:10.1002/ohn.70069)
Supplement: Supplementary file 2 — Supporting Information. [file OHN-174-57-s001.docx]

**Supplementary Document Legend:**

This supplementary file supports the primary manuscript with additional detail and transparency regarding methods, evaluations, and outcomes related to the systematic review and meta-analysis of the functional outcomes of TORS. It is divided into four sections:

**Section 1 – Search Strategy**
Provides a detailed account of the systematic literature search conducted across multiple databases and grey literature sources. It includes the full search syntax, the deduplication process, and database-specific results. A summary of manual and web-based searches is outlined.

**Section 2 – Risk of Bias Assessment**
Outlines the systematic assessment of methodological quality across included studies. Non-randomised studies were evaluated using the ROBINS-I tool, and randomised controlled trials with the ROB-2 tool. The section includes tables and figures summarising domain-specific and overall risk of bias judgments, enabling transparency in evidence quality appraisal.

**Section 3 – Extended GRADE Evidence Profiles**

Presents Extended Grading of Recommendations Assessment, Development and Evaluation (GRADE) evidence profiles for key outcomes comparing Transoral Robotic Surgery (TORS) versus Chemoradiotherapy (CRT). Each table summarises the certainty of evidence across five GRADE domains:

- Risk of Bias: Methodological quality of included studies
- Inconsistency: Variability across study results
- Indirectness: Applicability of evidence to the review question
- Imprecision: Certainty in effect estimates (e.g. sample size, confidence intervals)
- Publication Bias: Risk of selective reporting or missing studies

Each outcome is assigned an Overall Certainty rating: *High*, *Moderate*, *Low*, or *Very Low*. Tables are organised by outcome type and timepoint (e.g. swallowing function via MDADI at 3–6 months, gastrostomy tube dependence at 12 months).

**Section 4 – Comparative Analysis of Swallowing Function Outcomes**
Presents a comparative analysis of swallowing function outcomes in patients treated with Transoral Robotic Surgery (TORS) versus those receiving radiotherapy (RT) or chemoradiotherapy (CRT). Data are drawn from validated instruments, including MD Anderson Dysphagia Inventory (MDADI); Dynamic Imaging Grade of Swallowing Toxicity (DIGEST) and Modified Barium Swallow Impairment Profile (MBSImP); Functional Oral Intake Scale (FOIS). Time points range from early (2 weeks) to long-term (up to 3 years). Where available, p-values indicate the statistical significance of between-group differences.
